# Who cares for the carers? carerhelp: development and evaluation of an online resource to support the wellbeing of those caring for family members at the end of their life

**DOI:** 10.1186/s12904-023-01225-1

**Published:** 2023-07-20

**Authors:** Jennifer Tieman, Peter Hudson, Kristina Thomas, Di Saward, Deborah Parker

**Affiliations:** 1 Research Centre for Palliative Care, Death and Dying. CareSearch Director, Palliative and Supportive Services, College of Nursing and Health Sciences, Flinders University, Adelaide, South Australia Australia; 2 Centre for Palliative Care, University of Melbourne & St Vincent’s Hospital, Professor Vrije University, Melbourne, Brussels, Australia; 3 Centre for Palliative Care, University of Melbourne & St Vincent’s Hospital, Melbourne, Australia; 4 Research Nurse/Project Officer Centre for Palliative Care, St Vincent’s Hospital, Melbourne, Australia; 5 School of Nursing and Midwifery, IMPACCT University of Technology Sydney (UTS), Sydney, NSW Australia

**Keywords:** Caregivers, Palliative care, Websites, Bereavement, Digital health

## Abstract

**Background:**

Most people living with a terminal illness and approaching death will need the assistance of a non-professional carer such as a family member, friend, or neighbour to provide physical, emotional, and practical caring supports. A significant portion of these carers can feel overwhelmed, isolated and experience psychological and/or financial distress. Carers can have unmet information needs and information needs can change across the caring period.

**Methods:**

Guided by an Australian National Reference Group, this project undertook a multiphase set of activities to enable the development of an online carer resource. These activities included a literature review of key issues and considerations for family carers supporting someone with a terminal illness, a scoping scan of existing online resources, and interviews and focus groups with eighteen carers to understand their needs and context of caring. This information formed the basis for potential digital content. A web project team was established to create the information architecture and content pathways. User testing survey and usability assessment of the CarerHelp Website was undertaken to assess/optimise functionality prior to release. An evaluation process was also devised.

**Results:**

The literature review identified carer needs for practical and psychological support along with better education and strategies to improve communication. The scoping scan of available online resources suggested that while information available to carers is plentiful, much of that which is provided is general, disparately located, inadequately detailed, and disease specific. The eighteen carers who were interviewed highlighted the need for helpful information on: services, symptom management, relationships, preparation for death, managing the emotional and psychological burden that often accompanies caring, and support during bereavement. User testing and usability assessment of the prototype resource led to changes to enhance the user experience and effectiveness of navigation. It also highlighted a lack of awareness of existing resources and the needs of marketing and communication to address this problem.

**Conclusions:**

The project led to the development of an open access online resource, CarerHelp (www.carerhelp.com.au), for use by carers and families caring for a person who has palliative care needs. The web metrics demonstrate substantial use of the resources.

**Supplementary Information:**

The online version contains supplementary material available at 10.1186/s12904-023-01225-1.

## Background

Over 160,000 Australians are expected to die each year [1]. Almost all of these people will need the support of a family carer, particularly those who are living at home, with the family providing a critical care integration with the health professional team [2–4]. When people who are approaching the end of life are asked about what matters most to them one of the most common responses is a desire for their family to be supported including during bereavement [5]. This can also influence the terminally ill person’s discussions about place of care and place of death if the patient feels a burden to family and family carers [6]. While many carers identify positive aspects, approximately 30% of family carers may be psychologically distressed, many are isolated and suffer financially [7–9]. For nearly all family carers this will be their first experience of caring for someone who is at the end of life, and many say that it is overwhelming [10, 11].

Caring for a terminally ill person requires different information at different times [12–14]. Carers need information about how to recognise when death may be on the horizon, what to expect when someone is imminently dying, and where to get help and support. Carers who are more informed can be better prepared for their role helping them to feel less distressed, feel more competent and cope better during bereavement [15, 16].

The role of informal carers supporting terminally ill people living at home is increasingly being recognised as a significant policy and research issue. Integrated care, personalised care, and carer support have been seen as important in building comprehensive policy supports for palliative care provision [17, 18]. The World Health Organization (WHO) guidance on palliative care recognises the need to support the patient and the family and also acknowledges the need for a team approach to support patients and their caregivers [19].

Internationally the importance of unpaid carer to care at the end of life has been recognised. A review of UK Policy documents suggests that an evidence-based approach to policy priorities of integrated care, personalised care, and support for unpaid carers could enhance palliative care outcomes [18]. The Lancet Commission’s report on the value of death argues for a better system of death and dying where networks of care lead support for people dying, caring, and grieving [20]. In Australia, the crucial role of carers in palliative and end of life care has been acknowledged in national policies such as the 2018 National Palliative Care Strategy [21] and The National Safety and Quality Health Service Standards developed by the Australian Commission on Safety and Quality in Health Care [22].

Increasing access to information is a critical aspect of enhancing carer capability and knowledge and building community awareness around caring and carer contributions in supporting people at the end of their life [15, 23]. Digital solutions are also of increasing interest given their ability to provide continuous availability and ready access [10, 24–27]. However, family carers can find it difficult to access needed information in a timely way and health professionals can find it difficult to identify and recommend trustworthy resources for their patients and carers.

This paper describes the project and research underpinning the development and formative evaluation of the CarerHelp online resource. Research describing the evaluation of the resource post release and selected components of its development are reported elsewhere [28, 29].

## Methods

### Aim

The project aimed to develop high quality content for an online resource for those supporting a family member nearing the end of their life, by identifying:

- The core needs of family carers of people with advanced disease; and.
- Existing, high-quality web-based content and resources that focus on providing information to support family carers of people with advanced disease in Australia.

Existing resources were identified in view of the fact that the information needs of carers vary depending on timing, carer capabilities and disease progression. The project therefore intended to identify gaps and/or areas in which value could be added to support carers, rather than duplicate existing quality materials.

From the outset, the project was guided by the knowledge and experience of a highly engaged National Reference Group (NRG). The NRG comprised representatives from 20 peak bodies and institutions (all of which were relevant to the focus of the study (see Table 1) and met regularly throughout the project providing expert advice and feedback on different activities.

**Table 1:** CarerHelp National Reference Group: Role and Organisation

Co-Deputy Director, Centre for Palliative Care & St Vincent’s Hospital Melbourne	
Nurse Practitioner, Grampians Regional Palliative Care Team	
CEO, Australian Centre for Grief and Bereavement	
Policy & Projects, The Federation of Ethnic Communities’ Councils of Australia Inc (FECCA)	
National Policy Advisor, Policy and Programs, Palliative Care Australia	
CEO, Chronic Illness Alliance	
Senior Manager, Cancer Australia	
CEO, Lung Foundation Australia	
CEO, Neurological Alliance Australia (NAA) & Multiple Sclerosis Australia	
CEO, MND Australia	
Consumer Engagement Coordinator, Dementia Australia	
CEO, Parkinson’s Australia	
Stroke Connect Officer, The Stroke Foundation	
Professor of General Practice, Primary Care Clinical Unit, Faculty of Medicine, University of Queensland	
Manager – Ageing and Aged Care Project, National LGBTI Health Alliance	
General Manager for Consumer Health Services, Healthdirect Australia	
President of the Board, Congress of Aboriginal and Torres Strait Islander Nurses and Midwives (CATSINaM)	

### Study design

#### Project phases

The project comprised a multimethod approach incorporating:

- A *literature review* of the published evidence pertaining to the needs of family carers of patients with advanced disease.
- A national and international *scoping scan* for existing, online resources that provide information to support family carers of people with advanced disease in Australia.
- *Interviews* and *focus groups* with family carers to identify areas in which information was needed, relevant content, perceptions on using the internet and optimum ways in which content should be presented.
- *Content resource development* for delivery through a web resource.
- *Pre-release usability assessment* and *user feedback*.
- Usage from web metrics.

**Fig. 1:**
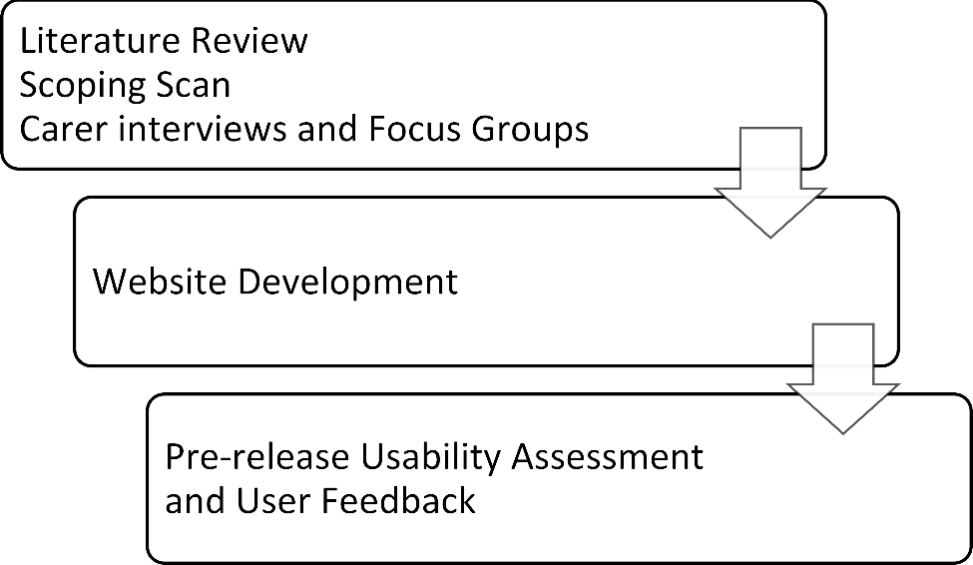
Schematic representation of project activities to develop the CarerHelp website

Each of the project activities contributed to the project aims of developing an online resource for carers, later named the CarerHelp website (See Fig. 1).

#### Literature Review

The first part of the project involved a rapid review of systematic reviews with a palliative care context [30]. This review was completed by the Centre for Palliative Care. The purpose of the review was to identify the core needs of family carers of people with advanced disease. Analysis comprised tallying the needs of carers reported in the results and discussion sections of each included paper along with the disease cohort being cared for. Results were classified into broader need and disease categories, where possible, and analysed descriptively using frequency histograms and heatmap matrices. The results informed the development of content for the online resource. Details pertaining to how the literature review was conducted are outlined in Additional File 1.

#### Scoping scan

In addition to the literature review, a national and international scoping scan was undertaken to identify suitable existing online resources. Websites providing information to family carers of adult patients who are diagnosed with an advanced life limiting disease such as cancer, dementia, neurological disease, heart/vascular disease, respiratory disease, motor neurone disease/ALS, kidney disease or liver disease were identified by members of the NRG and a search of related organisations. The main focus was on Australian websites however sites from the UK, USA, Canada, and New Zealand were also included. Each website was then reviewed for relevance and quality by senior researchers in the field of palliative care. Details pertaining to how the Scoping Scan was conducted are outlined in Additional File 2.

#### Interviews and focus groups

Focus groups and interviews were scheduled both face to face and via video conferencing to provide national representation. Potential participants were to be recruited through national carer organisations and disease peak bodies relating to common diseases such as cancer or heart failure (e.g. Cancer Australia and The Heart Foundation).

For inclusion in interviews/focus groups, participants needed to be either currently caring for someone with an advanced disease or had previously cared for someone who had died from an advanced disease. Interviewees were from diverse backgrounds. Analysis of interview and focus group data was undertaken by senior staff from St. Vincent’s Hospital. Interviews were recorded, transcribed and analysed for key themes. The focus groups and interviews were analysed using the five steps of thematic analysis recommended by Boyatzis [31]: (1) reducing the raw information, (2) creating a code (3) determining the reliability of the code (4) identifying themes within subthemes and (5) comparing themes across subsamples. Further details pertaining to how the Interviews and Focus Groups were conducted are outlined in Additional File 3.

#### Website development

A web content team was established to undertake the content development and design the architecture of the web resource. It comprised the investigators, digital lead, educational designer, and project officer. The approach to website development was based on the Centre for eHealth Research (the CeHRes) iterative model that had mapped the research and developmental activities involved in developing eHealth applications from concept definition through development to summative evaluation [32]. It highlighted the importance of needs assessment, meaningful user content, and processes to test design and function as well as evaluation after release. This approach had been used in previous developmental work undertaken by the web team [33], The preliminary content architecture was based on the literature and scoping scan reflecting carer perceptions of information needs and knowledge gaps Changing information was also seen as a critical function to enable carers to navigate easily to useful information and led to the development of a series of carer pathways. Technical issues addressing accessibility compliance, page templates, content formats and graphic styles were also negotiated.

#### Pre-release usability assessment and user feedback

Members of the National Reference Group and carers involved in interviews and/or focus groups were provided with the opportunity to visit the site prior to release and invited to provide comments and feedback as part of informal user testing. Given that usability of digital resources is identified as a key component of good practice in the development of digital applications, formal usability testing was undertaken to identify design or build errors [34]. All carers who had participated in the interviews were invited to be involved in a user testing exercise. This involved the review of the test site and completion of an online feedback survey (see Additional File 4: CarerHelp Website User Testing Review Form).

Attendees at the national Australian palliative care conference were also invited to participate in a health professional review of the final version of the web resource just prior to its release (See Additional File 5: The Australian Carer Toolkit for Advanced Disease: Survey).

In a companion exercise, a PhD student undertook an independent usability activity with carers of the online resource. The PhD student conducted usability tasks with six current or previous carers of a palliative care patients. Six participants were given tasks to perform and asked to use the think aloud protocol. Test sessions lasted an average of 70 min. Full details of the usability processes can be found in Additional File 6: Report of Usability Test Findings of the Australian Carers Toolkit.

#### Usage patterns

Web metric collection was planned to determine usage rates and page pattern usage. Success metrics of 250 users and 750 page views of the online resource each month after release had been specified in the original grant proposal.

## Results

### Literature Review

**Fig. 2:**
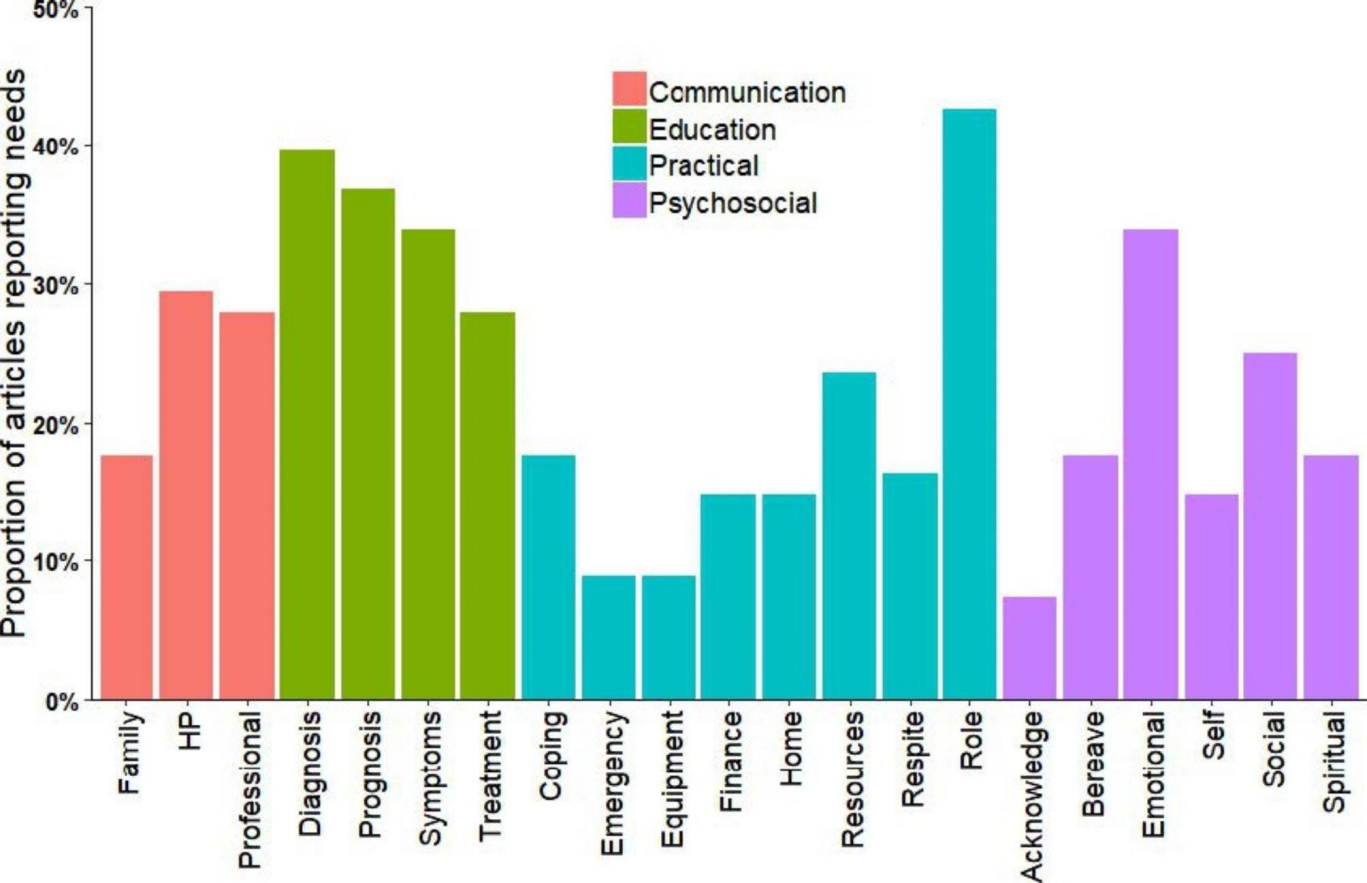
Common topics organised into dominant categories

Results from the database search identified 2,674 potentially relevant articles, of which 68 met the inclusion criteria for the review. Information regarding reported carer needs and disease cohort was extracted from each article. Figure 2 highlights reported carer needs against four categories of carer needs. The key findings from the literature review are summarised below.

- A wide range of carer needs were reported across diseases. Needs were largely representative of four main categories: Communication, Education, Practical, and Psychosocial support. The most commonly reported needs were from the Education category.
- Disease cohorts were from the following main categories: Advanced disease (non-specific), Malignant, and Non-malignant. The majority of papers investigated carer needs in the Advanced disease (non-specific) category.
- The single most highly reported need across all diseases was “practical support” with respect to the role of the carer. The top four reported needs (“symptom management”, “carer role”, and education about “prognosis” and “diagnosis”) were documented most frequently in the “cancer” and “end-of-life” disease cohorts.
- Carers looking after people from motor neuron disease, chronic obstructive pulmonary disease, cancer, palliative care, glioma, and end-of-life disease cohorts reported the widest range of needs spanning across all four need categories.

Figure 2 highlights reported carer needs against four categories of carer needs.

### Scoping scan

In total, 33 websites were included in the scoping scan which sought to identify relevant content and resources for potential inclusion in the CarerHelp resource. Many of the reviewed websites did have relevant resources. Of these, four were cancer related, six were disease specific (non-cancer) and 23 were generic (not related to any specific disease). Key findings from the scoping scan were that:

- Information exists on many issues relating to carer activity across a variety of websites. However, the difficulty for carers is finding the information in one place.
- The disease specific sites do not include much information on choosing site of care (e.g. home, hospital, aged care facility), however this is covered well by palliative care sites. If you are caring for someone who is not linked in with palliative care, then you would be unlikely to access this information.
- Coping with emotions and difficult conversations as a content area is not frequently covered even though it is seen as a priority area for carers. Similarly, changing roles and relationships is not frequently covered by disease specific sites.
- There is a general lack of information that is specific to Culturally and Linguistically Diverse (CALD) populations, Lesbian, Gay, Bisexual, Transgender, Intersex and Queer (LGBTIQ) populations or Aboriginal and Torres Strait Islander (ATSI) populations.
- While many sites offer videos, they are of varying quality and content.
- While death and dying is covered by many sites (generic and disease-specific), more detailed information on death and dying from a carers’ perspective appears to be an information gap.

This highlighted the needs for a comprehensive resource that brought together information for carers of those with advanced disease to enable direct access to relevant resources.

### Carer interviews/focus groups

Eighteen carers from around Australia (see Appendix 1) who were currently caring for someone with an advanced disease or had previously cared for someone who had died from an advanced disease were involved. Key care considerations with illustrative comments are detailed in Table 2 below. The information was used to design resources and content that meet the needs of carers.

**Table 2:** Key Care Considerations

Theme	Data/Quotation
General advice for carers	‘Planning questions ahead of time (for medical appointments). Taking notes or recording conversations.’ ‘Try time out activities together (ways to relax while still caring). Things that the person can still enjoy (going to a concert of live music, sitting on the beach).’
Managing the emotional and physical burden of caring	‘Looking after yourself may just be about living day to day and utilising as much assistance as you can to get through. When care goes on for longer than expected you may start to feel that you need a different strategy that actually ensures you get time out and actively do activities that may assist.’ ‘Stay connected with friends and family.’
Information for carers on how to prepare for the death of the person cared for	‘I wanted more preparation for the death – even though I have seen others die, I have not been the carer before and I was surprised by the impact.’ ‘I needed more information on what symptoms were due to the illness and needed treating (medication adjustment) and what was part of the dying process that may not need to be treated.’
Information on services available to carers and how to find/use them	‘The services are not as good in regional areas, they only do overnight for crises situation or symptom management.’ ‘Understanding what palliative care is and to make contact early – provide emotional support as well as practical and medical.’
Information on support services available to carers following the death of the person cared for	‘I wasn’t prepared for the grief. I found it helpful to read about death from other carer’s experiences.’ ‘My two siblings and I made contact with psychologists prior to Mum dying and had 10 sessions each so that we could talk thru the dying process and then after death.’

### Website development

Following the literature review, scoping scan and carer interviews and focus groups, a mapping of the content areas was completed. This showed that there was a challenging breadth and diversity of information needs and content resources. Findings from the literature review and from the carer interviews confirmed that carers felt they needed more information on understanding the practical and emotional issues in supporting a dying person. Existing resources also tended to be disease specific or generic in terms of carer activities. The project activities also showed that there was less material available that directly supported carers preparing for dying and managing the dying period and this did not support carer preparedness to engage in the challenging aspects of direct care at the end of life.

The content developed in the first three activities informed a summary proposal of content and resource packets for use in the proposed website for discussion with the National Reference Group. Feedback from this group supported the need for a repository of relevant content and online resources dealing with information needs, ways to support carers navigating to information and resources relevant to the proximity to death and dying, and different formats in which carers could engage with content.

The Web Content Team was tasked with translating the content resources and navigation considerations into a menu architecture and visual representation that would then be reviewed and critiqued by the National Reference Group. The Web Content Team scheduled a series of day meetings during the four month production schedule to examine the organisation of content and approaches to recognise different experiences and carer journeys. In addition, weekly video conferences were maintained.

These meetings led to three specific solutions which were confirmed with the National Reference Group. First a Carer Library was needed to collate existing quality resources and provide carers seeking a specific resource with direct navigation to relevant resources. The Carer Library would also contain specific project content developed by the project team on topics that were not well addressed within existing materials. This content would be made available as PDFs to facilitate sharing of resources within the family or from health professional to the carer.

The second structural component was the introduction of a set of carer pathways that organised resources around knowledge and information needs for different time points including the death and dying period. This bundled resources appropriate to differing information needs over time. The five pathways proposed were:

- Pathway 1: When someone needs care.
- Pathway 2: Caring when death is a possibility.
- Pathway 3: Preparing for dying.
- Pathway 4: When the person is dying.
- Pathway 5: After caring.

The third structural element was to provide content in different content formats to support different user preferences. Content was to be shaped into modules and videos as well as web pages. Each pathway included video or module content as well as links to project PDFs and profiled resources relevant to the specific pathway.

The project team then identified content sheets needing to be developed, videos and modules to support he proposed pathways. Options for the organisation of the online site were formulated and tested for their feasibility. A graphic designer was recruited to produce brand and logo solutions.

The online resource structural elements, proposed web architecture and graphic design options were reviewed by the project team and by the National Reference Group throughout the development phase. Information resources and web content was reviewed against readability standards and web resources were checked for accessibility compliance.

The National Reference Group was also involved in the review of options for a resource name, electing to name the resource CarerHelp. The logo and brand design was then deployed for use within the online resource and for promotional and marketing resources.

### Pre-release Usability Assessment and user feedback

#### Carer user feedback

Seven carers provided comments and feedback on the CarerHelp resource as part of user testing. A further six people associated with carer organisations also provided feedback. Participants in the usability exercise were naive to the project and recruited through carer and palliative care bodies and organisations. The user testing exercise involved carers who had participated in the interviews and accepted the invitation. In addition, members of the NRG were also involved.

A number of minor issues relating to spelling or navigation were identified and corrected. Users also described their views about the website. The major feedback themes with examples of carer comments are described in Table 3 below.

**Table 3:** Major Themes and Carer Comments from the User Testing Exercise

Theme	Data/Quotation
Information was comprehensive	‘I wish I did have this list (Question Prompt List) when my parents were at their end of life stage. As an outsider we don’t know what to expect and what questions to ask.’ Very thorough. Wish I had some of this 10–15 years ago.’
Hearing other carer views	‘It is interesting to read other people’s stories to know that you are not alone.’
Limitations of being online	‘Not everybody has access to a computer or knows how to use a computer.’ ‘I think it’s extremely comprehensive, it could be a little bit overwhelming. It’s easier for people who are computer literate.’
Carer pathways were useful	‘Caring means you have little time to browse; so if you make it clear that these separate stages can be accessed in one more click, it saves hunting.’ ‘Navigation is very simple and fast. It is easy to understand, and it acts as the needed gateway to work through the various specialty areas. As I signaled earlier, the care pathways are key and very sensible.’

#### Health professional user feedback

Twenty-eight survey responses were received from people attending the national conference. Twenty-three respondents were health professionals, two were family members, one a volunteer, and two did not specify. Twenty-five respondents indicated they would refer someone to CarerHelp while three did not reply to this question. Respondents also provided suggestions on how to use the resources as a health professional and on how to promote CarerHelp.

#### Usability Assessment Exercise

The report provided to the project team indicated that the participants found the Carers Toolkit to be a generally useful and relevant online resource that will be acceptable to the targeted audience. Participants found the content to be extremely valuable especially the practical tools and guidance supporting care during the intermediate stages before the terminal phase. The majority of issues identified by this usability evaluation were related to the navigation into and around the internal pages of the Toolkit including text descriptors and landmarks whilst the language used in the protype was highlighted as a problem for some carers.

Issues and concerns identified in user testing and the usability assessment were addressed prior to release. Modifications relating to reading levels, limited diversity in imagery, font size and navigation processes were corrected prior to release.

#### Usage patterns

The CarerHelp website was released in October 2019. The post release web metrics show sustained use of the resources in excess of the specified metrics of 250 users and 750 page views per month. From the launch date until the end of the project period on 30 June 2020, there have been 5,701 users, 25,214 webpage views and 7,881 web sessions. On average there were 633 web users, and 2802 web page views each month.

## Discussion

The carer population is diverse. Carers experience different information needs at different times. Therefore, information resources need to be relevant and meaningful as well as accessible. CarerHelp provides online resources that aim to address the core needs of the family carers of people with advanced disease.

Developing online resources for carers is a complex undertaking. Individual carers will have specific information needs reflecting the particular circumstances of the person for whom they are providing care. There is a growing literature that addresses the value and processes associated with effective web design and user engagement [35–37]. These considerations may be of even more importance in supporting family carers of those coming to the end of life given the emotional impacts and time pressures experienced in such caring. Individuals also differ in their cultural and language backgrounds as well as their digital capabilities making it difficult to create meaningful resources that address all needs and all skills [10]. The comprehensive approach utilised in CarerHelp enabled triangulation between available resources, evidence of need through review of research studies and carer interviews, and utility to the end user through usability exercises.

Previous studies have highlighted that in palliative care, family caregivers need to be prepared for the patient’s death [38, 39]. Similar considerations had been reported by Scott et al. in their development of an Irish website designed to help carers feel more prepared to manage the challenges associated with caring for a relative or friend receiving palliative care [10]. The research underpinning content for use in our website not only identified useful material but was instrumental in identifying a lack of specific materials about the reality of preparing for and managing death and dying. This enabled a focus for resource development that spoke to these particular needs and meant that included resources were designed to support carers at the appropriate point of need.

The usability and user testing contributions should not be underestimated in sharpening the focus of the contents included, the language and the nature of the guidance being provided for carers. This speaks to the importance of ensuring that sufficient time and resources are allocated to the initial consultations and content development and review processes. It is also worth noting that many of the carers involved with the program have continued an involvement in promotional activities and in ongoing feedback about the project.

As well as consumer feedback, an informed approach to web design processes addressing issues such as content standards, accessibility, language levels and consumer accreditation (such as Honcode and Healthdirect) are important in demonstrating the credibility of online resources for a potentially vulnerable population. Such strategies also form part of translation and implementation approaches as they provide context and endorsement of the quality of the content, while web effective approaches enable direct retrieval and utility by a broader range of users. The CarerHelp marketing strategy seeks not only to build brand awareness but also to utilise broker agencies including specialist palliative cares services, care organisations and consumer groups who provide a bridge to carers who may not have had direct access or interest in online resources.

Web metrics highlight the value of the resource with more than 600 visits to the online resource each month. The usage data indicates an ongoing interest in the resource and patterns of page views and visits highlights that there is a willingness of carers to engage with content that can seem to be confronting such as the death process and the care needs during dying.

Some issues identified in the project activities continue to drive improvements. Maintaining currency of evidence to inform guidance is crucial if the ongoing value of the resource is to be realised. A companion review undertaken by the project team examined the commonly expressed needs of family carers of people and preferred knowledge sources [40]. Findings from this review are already informing improvements to the project resources. Carer interviews and the literature review showed a diversity of needs and diversity among the carer population. While some materials addressing cultural diversity and geographic impacts had already been included, more focused work is currently underway to expand the site with dedicated resources for different cultural resources and for Aboriginal and Torres Strait Islander peoples.

There are also remaining questions about to identify and engage with carers who may not have high levels of digital health literacy. The WHO has already recognised the role of information and communication technology (ICT) in health. The release of a 2020–2025 strategic plan that promotes equity, affordability and access reinforces the need for a social justice approach to digital health developments [41]. Negotiating mechanisms to enable dissemination to marginalised carers will be critical to ensure equitable care provision. Support workers, health professionals and social care providers will be critical in sharing the information and resources. Technologies such a VoiceOver, Speak and Select or Speak screen can make words more accessible to non-English speakers or those who prefer to have the text spoken rather than read it. Carers could also potentially benefit by online support groups such as those trialled for dementia carers could be useful [42].

It is also clear that the role of health professionals in different settings is important in supporting carers and in guiding carers to valuable resources. This will require targeted and sustained promotion and communication not only to build awareness but to understand how best to incorporate CarerHelp resources into services and into health professional interactions with palliative care patients and carers.

### Limitations

While a comprehensive literature review was undertaken there may have been issues that were not identified in the review. While the development of the website was guided by the CeHRes Framework, the project was not designed as a formal research study and did not have an underpinning theoretical position. However, at each stage, the work was referenced back to user perspectives, best practice approaches to web development and both formative and post release evaluation. Although a diverse group of carers were engaged with in the consultation period there may be other needs and concerns that have not been recognised. Online information is obviously restricted to those that have the ability to engage with digital resources which may limit the uptake of available resources.

## Conclusions

Carers of people with advanced disease facing death have ongoing and changing information needs. Being able to identify these not only through the literature but through consultation with carers themselves and to confirm through usability and user testing is important to ensure not only trustworthy resources but resources that are relevant to the carer group themselves. The project described in this article led to the development of an open access online resource, CarerHelp (www.carerhelp.com.au), for use by carers and families caring for a person who has palliative care needs. The web metrics demonstrate substantial use of the resources.

## Electronic supplementary material

Below is the link to the electronic supplementary material.

Additional File 1. The Australian Carer Toolkit for Advanced Disease: Literature Review.

Additional File 2. Summary Report: The Australian Carer Toolkit for Advanced Disease: Scoping Study.

Additional File 3. The Australian Carer Toolkit for Advanced Disease: Focus Groups and Interviews.

Additional File 4: CarerHelp Website User Testing Review Form.

Additional File 5: The Australian Carer Toolkit for Advanced Disease: Survey.

Appendix 1. Interview and Focus Group Participants

Additional File 6. Report of Usability Test Findings of the Australian Carers Toolkit.

## Data Availability

The datasets generated and/or analysed during the CarerHelp Project are not publicly available due to personal and detailed information that could potentially identify participants, but are available from the corresponding author on reasonable request.

## List of Abbreviations

ATSI: Aboriginal and Torres Strait Islander
ACSQHC: Australian Commission on Safety and Quality in Health Care
CATSINaM: Congress of Aboriginal and Torres Strait Islander Nurses and Midwives
CALD: Culturally and Linguistically Diverse
EAG: Expert Advisory Group
FECCA: Federation of Ethnic Communities’ Councils of Australia Inc.
HON: Health on the Net
LGBTIQ: Lesbian, Gay, Bisexual, Transgender, Intersex and Queer
NRG: National Reference Group
NSQHS: National Safety and Quality Health Service
NAA: Neurological Alliance Australia
WHO: World Health Organization
