# Supplementary material for: Who cares for the carers? carerhelp: development and evaluation of an online resource to support the wellbeing of those caring for family members at the end of their life
Source: BMC Palliat Care. 2023 Jul 20;22:98. doi: 10.1186/s12904-023-01225-1 (PMC10357776; doi:10.1186/s12904-023-01225-1)
Supplement: Supplementary file 1 — Additional File 1. The Australian Carer Toolkit for Advanced Disease: Literature Review. [file 12904_2023_1225_MOESM1_ESM.pdf]

The Australian Carer Toolkit for Advanced Disease:

Focus Groups and Interviews

Summary Report

February 2019

PREPARED BY:

KRISTINA THOMAS

DIANNE SAWARD

PETER HUDSON

THE CENTRE FOR PALLIATIVE CARE

ST VINCENT'S HOSPITAL MELBOURNE & THE UNIVERSITY OF MELBOURNE

Background

As part of the *Australian Family Carer Toolkit for Advanced Disease* project, focus groups and/ interviews family carers were undertaken to assist with the development of appropriate content for the proposed online resource.

Aims of focus groups and interviews:

The aims of the carer focus groups and interviews were to:

1. Discuss the draft content areas of the Carer Toolkit (Attachment 1) and obtain feedback from carers on the relevancy and structure of the content areas and any perceived gaps.

2. Discuss barriers and enablers on using the internet to find information and preferences regarding the format they want the content presented (for example: written, spoken, videos, case studies, short exercises).

Method

Qualitative interviews and focus groups were conducted with current or bereaved carers of people with advanced disease.

Our inclusion criteria were: A current or bereaved carer of an adult (18 years or older) with an advanced disease; at least 18 years of age; able to speak English; willing to participate in a telephone interview or attend a focus group.

We invited members of the National Reference Group committee, set up to guide the *Australian Family Carer Toolkit for Advanced Disease* project, to assist in recruiting participants. A number of members volunteered their organisation as a means of advertising the study to potential participants. We recruited participants through Cancer Australia, Dementia Australia, Carers Australia, and LGBTI Health via advertising the study in their newsletters (LGBTI Health and Cancer Australia) or as an email mail out to carers that met our inclusion criteria (Dementia Australia and Carers Australia). Interested carers contacted the Centre for Palliative Care directly. A researcher contacted the carer, checked they met the inclusion criteria, and obtained their location. Once we had at least four interested carers in the one location, a focus group was organised and details sent to the carers. Only one focus group was organised for Sydney and conducted by two researchers from the Centre for Palliative Care. For all remaining interested carers a telephone interview was offered at a convenient time. Interviews were conducted by one of two researchers from the Centre for Palliative Care.

A semi-structured interview schedule (Appendix A) was developed by the Project Management Committee and reviewed by the Executive Committee. The same interview schedule was used for the focus group and interviews. Interviews were recorded and transcribed and analysed for key themes. The focus groups and interviews were analysed using the five steps of thematic analysis recommended by Boyatzi[1] [54]: (1) reducing the raw information, (2) creating a code, (3) determining the reliability of the code, (4) identifying themes within subthemes and (5) comparing themes across subsamples.

Results

Eighteen carers participated in the study. Five attended a focus group (in Sydney) and the remaining 13 were involved in a semi-structured interview over the telephone. Demographic information about the participants is outlined below:

- 6 participants were male and 12 were female
- Participants were caring for someone with a diagnosis of cancer (n=7), dementia (n=11), and someone with both HIV and cancer (n=1)
- The age range of participants was from the early 20's to the late 80's with the majority in their 60's
- 7 participants were current carers and 11 were bereaved carers
- 1 carer represented the LGBTI community
- 1 carer represented the CALD community
- 6 participants were from rural or regional areas and 12 were living in a metropolitan area
- Participants were from 5 states including NSW (n=10), Victoria (n=4), Tasmania (n=1), Queensland (n=1), South Australia (n=1), and Western Australia (n=1)

A number of themes arose from the qualitative data related to: the way participants use the internet; preferences on terminology; their preferred format of information on the internet; and content areas that participants were interested in.

The way participants use the internet

The majority of participants (16/18) felt comfortable using the internet and seeking information about the disease or about the caring role from the internet.

Preferences on terminology

The majority of participants were comfortable with the term 'Carer', only two preferring 'support person' or 'companion'. They all liked the suggested names for the new resource, 'Carer Help' or 'Carer Toolkit' with the preference for one name over the other being split evenly amongst the participants. Participants commented that the term 'Toolkit' sounded a bit 'more positive' but also a bit 'unfriendly' and 'technical'. On the other hand, 'Help' was viewed as a 'bit negative' because it implies you need help. A few other names were suggested such as Carer lifeline, Help4Carers, Carer Guide, Carer Tools, and Tools4Carers.

Preferred format of information on the internet

Many participants commented they just wanted to go to the internet and get their information quickly. Others reported they preferred to make a connection through videos of carers talking or something similar. A few participants commented that cartoons would be a good way to deliver sensitive information and lighten the mood. Some participants were open to undertaking educational training modules on how to care or how to look after themselves. Others did not like this idea as they preferred to quickly look up what they needed to on their own.

Other specific comments on the format of the proposed website are listed below:

- A pop up question, such as ‘How are you feeling today?’
- Lots of carer videos (as long as they are short), cartoons and graphics.
- Case studies
- Information on topics to read
- A 10 minute tip on ….
- Educational learning modules on caring
- Something that was easy to navigate and simple to understand
- Search topics listed alphabetically
- The ability to highlight sections that they want to read again or put it in a separate folder to read later
- Keep it light-hearted since it is a serious topic
- Factual information
- The ability to print off information in an easy to read format
- Simple, friendly writing
- No acronyms
- Dot points which are clear and really simple

Content areas that participants were interested in

When participants were asked what sort of information they would be looking for (without being prompted with our modules – Attachment 1) they mostly suggested symptom management or disease specific information. A couple mentioned information on end of life or assistance finding services or aged care facilities. However, many thought that once they were on the site they were open to finding other information or having a pop up with suggested pages to read. Many of the bereaved carers mentioned the need for information and strategies on caring for themselves but said that this was difficult to think about while actually caring.

Once participants were prompted with our content suggestions (see Attachment A: Modules), they had a lot of comments on information and content that they thought was important and gaps that we had not included in our draft content areas. The main comments on content have been grouped by theme and are listed below:

Content theme: The family context (and broader social context)

Some participants commented that the relationship with the person you are caring for is not always a good relationship. Similarly, a participant highlighted that as a carer you may have to deal with family members that you have a difficult history with.

> *'It is not always a good relationship with the person you are caring for.'*

> *'Sometimes it is a complex situation if you are the primary carer and other family members are estranged to the patient or to you. How to relate to family members about important developments in these complex family situations.'*

Participants also highlighted difficulties with the social context when someone has an advanced disease. Specifically, how to deal with negative or avoidant reactions of others and the social isolation of going through an experience that others can't relate to. One person wanted information on how to encourage others to feel more comfortable so that they would visit.

> *'How to help and encourage others (friends/family) to visit the patient and feel comfortable? How to deal with the reactions of others as it can be humiliating and hurtful to see others treat your partner differently?'*

> *'Social isolation and loneliness – unable to talk to friends because they don't understand what you are going through. Friends who are unable to cope with it.'*

Content theme: General advice on how to be a better carer

Participants wanted general advice on how to be a better carer. Some suggestions were how to deal with boredom, how to improve the quality of life of the patient, guidance on medications and symptom management, planning ahead for appointments, planning ahead in relation to practical affairs, and practical care. Carers made a lot of requests for information on management of specific symptoms or on particular care tasks. Requests for ways to assist carers be more organised and plan ahead were also made by a few carers.

> *'Planning questions ahead of time. Taking notes or recording conversation.'*

> *'Find a carer mentor – who has been through it before.'*

> *'It would be good to have an education section as there are so many untrained people that have responsibility caring.'*

A number of carers spoke of the boredom of caring for someone with an advanced disease both for themselves and for the person they were caring for. One person suggested trying to focus on the things

that the patient could still do and enjoy, particularly if they were things the carer and patient could enjoy together (e.g. listening to music, sitting on the beach).

'Made my life around what my 'husband' could still do and enjoy.'

'Try time out activities together (ways to relax while still caring). Things that the person can still enjoy (going to a concert of live music, sitting on the beach).'

Content theme: Positives of caring

Carers commented that there were also positives to the caring role. A few carers commented that they learnt many new skills which they could then use in other ways after the person had died, for example in volunteering or new employment.

Content theme: Location of care

A strong theme for many participants was where to care for the patient and how to deal with decisions around changing that location of care. They acknowledged that this was a big decision and that there were often complex feelings attached to the decision. Many participants who were caring for someone with advanced dementia also wanted more information on finding appropriate aged care services.

'It was a difficult decision to put her in care but it was the right decision. I was struggling and my health was deteriorating. Listen to those around you when they tell you that it is time.'

'Accepting that respite / aged care were the best option. Coming to terms with that.'

Content theme: The experience and emotional response

Many of the participants reported feeling exhausted and reported negative emotions such as depression, anxiety, grief and trauma. There was pressure in being responsible for the person's care constantly. Some spoke of the trauma of the intense but short caring experience while others spoke of the exhaustion of the caring experience that lasted years.

'I was so burnt out – overnights were exhausting. Then working during the day.'

'I experienced depression and anxiety. When the patient is depressed, it feels contagious'.

'Being the primary support person means the patient often does not want you to leave – pressure to be there all the time. I worry if I am not there.'

'Patient and carer are fused together but people only interested in patient's needs. We need to be thinking of the interaction between both – how one affects the other.'

Content theme: Looking after yourself

Information on looking after yourself (as a carer) was considered by most participants as very important, although some acknowledged that they found it very hard to do themselves. Participants agreed this section should be a feature on the website and highlight that carers are in a high risk group (both emotionally and physically) and to plan for the future (after caring finishes). They came up with many strategies that they think would be useful to other carers in looking after themselves throughout the caring experience including:

> *'Stay in some form of work if the carer wants to (as this provides stimulation, income, and it will be easier to return to work in the future).'*
>
> *'Plan ahead for when caring finishes and don't give up all your links/networks. Try to stay connected or it is much harder later.'*
>
> *'Looking after yourself may just be about living day to day and utilising as much assistance as you can to get through. When care goes on for longer than expected you may start to feel that you need a different strategy that actually ensures you get time out and actively do activities that may assist.'*
>
> *'Stay connected with friends and family.'*
>
> *'Get support for yourself. Set it up early so you have an established relationship during tough times/bereavement. Mental health plans through GP.'*
>
> *'Planning in the short term but also in the long term. Think about what you will need down the track.'*
>
> *'Using laughter and humour to help you get through it.'*

Content theme: Information on and preparation for death

Many bereaved participants spoke about how they found the death confronting and did not feel prepared enough for the death and the lead up to it. In general the comments related to wanting more information about how to know when death was close, what happens when someone dies, and what to expect in the time after death.

> *'I wanted more preparation for the death – even though I have seen others die, I have not been the carer before and I was surprised by the impact.'*
>
> *'I wished I had more experience on the end stage and caring for someone at home during that stage.'*

'I needed more information on what symptoms were due to the illness and needed treating (medication adjustment) and what was part of the dying process that may not need to be treated. Very confusing in the context of dementia.'

Other issues that arose, included confronting your own death, how to know when to let go, how to ease the person's suffering as they are dying, dealing with the transition from being a carer to a grieving person, how to talk to the person about death and dying, and understanding that advised time frames are not always accurate. One participant also noted that pre-planning the funeral and using a checklist was helpful to manage the practical aspects of death.

'If you are an older carer you are also confronting the inevitability of your own death.'

'When is it time to accept that they are going to die – try to stop treating them, medicating them, feeding them (if the patient is not wanting food). Hard to let go when your focus has been to keep them alive for a long time.'

'What happens to the body and who is caring for it? We needed to know where 'he' was and that he was being cared for and respected.'

'How to talk to your daughter (loved one) about them dying? And about how they might be feeling through this process and what to say to them. Feeling useless.'

Content theme: Services and financial support

Identifying external services and supports that are available in different regions was also commented on by many participants as important. In general, participants from rural/remote areas noted the lack of home care services. There were comments that navigating the system was frustrating and specifically understanding the various financial supports and knowing what to ask was very difficult. There were a couple of comments on needing more information about palliative care and how to access it. Participants made suggestions for the online resource to include links to online support groups, information on how to navigate various financial supports (NDIS, aged care pension, Centrelink), including helplines, registering with 'my aged care' and obtaining 'guardianship', and how to access services that assist in the home and provide equipment in the home.

'The services are not as good in regional areas, they only do overnight for crises situation or symptom management'

'How to navigate Centrelink, NDIS, aged care pension. Which is best? When do you swap between them? Knowing what questions to even ask?'
'Register for My Aged Care early'

'Understanding what palliative care is and to make contact early – provide emotional support as well as practical and medical.'

'Include helplines. Don't be afraid to call them.'

Content theme: Bereavement

Most of the bereaved carers that participated commented on bereavement saying they were not prepared for the experience of grieving what was to follow. For a few, there was some form of regret or guilt that they continued to ruminate about. One commented that information on organ donation would have been helpful before the death and another recommended making contact with a mental health professional before the death so that post death there was someone to talk to.

'During bereavement, agonising over details that led up to death (and what if…?). Regret. Guilt.'

'I wasn't prepared for the grief. I found it helpful to read about death from other carer's experiences.'

'My two siblings and I made contact with psychologists prior to Mum dying and had 10 sessions each so that we could talk thru the dying process and then after death.'

Limitations

As this sample was primarily carers of people with dementia or carers of people with cancer, the results may be biased towards the experiences and needs of those populations. Similarly, this small sample of 18 Australian carers may not reflect the experiences of all carers, particularly as this sample was biased towards anglosaxon Australians.

Conclusion and Recommendations for Carer Toolkit Project

The interviews and focus groups confirmed high levels of emotional and physical burden for this population of carers. There seemed to be a very strong need for further information on preparation for death and what to expect as death approaches. Other priority areas included information around how to be a better carer (or strategies to improve the experience for carers and patients) and around support services (including financial services) to assist this process. Location of care and how to navigate the aged care sector was also an area of need for these participants however this may be influenced by the fact that a large proportion of participants were carers of people with dementia.

Implications for Palliative Care

The remit of palliative care includes carers and families of people living with an advanced disease. Therefore results of the interviews which related to the needs of this population may be useful for the palliative care sector. Palliative care services are already aware of the large burden on carers and do attempt to meet the needs of carers. We recommend that palliative care continue its focus on carer needs and specifically around providing information and support to carers around preparing them for the death of their partner, relative or friend and what to expect as death approaches. The availability of in-home support services (or lack of them in rural and regional areas) and how to access them is something that needs review.

References

1. Boyatzis RE: **Transforming qualitative information: Thematic analysis and code development**: Sage; 1998.

Appendix A. Interview Schedule for focus groups and interviews

Thank you for taking the time to speak with me today. It is really important to us that we get your views on this website that we are developing. Having your involvement will assist us to ensure that we have the most relevant content and format for the website. Firstly, I am going to present you with some content areas that we are planning to include in the website. We have gathered this information from previous research, from looking at what information is already available on the internet, and from our expert panel. Please feel free to say as much or as little as you like.

Questions

1. In your time supporting your partner, relative or friend, when would you have gone in search of information and what sort of information were you looking for?
2. Thinking about your experience caring for someone with an advanced disease, what do you wish you had known? What do you wish someone had told you about?
3. From the topics listed, please comment on which topics you would have liked more information on as person involved in the care of someone with an advanced disease? (top 3)
4. Are there any topics that are not listed, that you believe should be included in the website? Why?
5. Are there any topics that are listed, that you think should not be included? Why not?
6. Thinking more broadly that just information needs – what would have made being a carer for someone with advanced disease easier?
7. How do you feel about the wording that we have chosen to use? Is there anything that you don't like or find inaccurate? Specifically so you identify with the term 'carer'?
8. Thinking about your own experience, how do/did you obtain information related to providing care to your relative, partner or friend?
9. Have you obtained information from the internet related to providing care to your relative, partner or friend? If so, what sort of information? Was it helpful?
10. If you have not obtained information from the internet related to providing care to your relative, partner or friend, why not? Would you consider using the internet in the future to obtain this information?
11. Do you trust the information you get on the internet? If not, would you trust the information of a website that someone you trusted referred you to (such as a GP or health professional)?
12. If you were searching the internet for information related to providing care to your relative, partner or friend, what words or terms would you use? Where would you start?
13. Thinking of the format that you like your information to come in (that is, written, video of a health professional talking, video of a patient or family member talking, explanatory pictures or diagrams etc), tell me about your preferences? What do you like? What don't you like?
14. When you go to a website, would you prefer to have topics listed and find what you need yourself or would you prefer some assistance (pop-ups with suggestions for other topic areas, pop ups asking you if you would like assistance to find information)?
15. We need to name the website, what do you think about the name Carer Help as an option?
16. Is there anything else that you would like to add?
