# Supplementary material for: Who cares for the carers? carerhelp: development and evaluation of an online resource to support the wellbeing of those caring for family members at the end of their life
Source: BMC Palliat Care. 2023 Jul 20;22:98. doi: 10.1186/s12904-023-01225-1 (PMC10357776; doi:10.1186/s12904-023-01225-1)
Supplement: Supplementary file 2 — Additional File 2. Summary Report: The Australian Carer Toolkit for Advanced Disease: Scoping Study. [file 12904_2023_1225_MOESM2_ESM.docx]

| **Demographics** | **N (%)** |
| --- | --- |
| Gender of Carer:  Male  Female | 6 (33%)  12 (66%) |
| Illness of patient:  Cancer  Dementia  Cancer and HIV | 7 (39%)  10 (55%)  1 (6%) |
| Age of Carer:  Under 30 years  30-50 years  50-70 years  Over 70 years | 1 (6%)  1 (6%)  12 (66%)  4 (22%) |
| Caring status:  Current Carer  Bereaved Carer | 7 (39%)  11 (61%) |
| Location:  Rural/regional  Metropolitan | 6 (33%)  12 (66%) |
| Diversity:  Identifies as LGBTI+  Identifies as culturally diverse | 1 (6%)  1 (6%) |

Appendix 1. Interview and Focus Group Participants
