# Supplementary material for: Who cares for the carers? carerhelp: development and evaluation of an online resource to support the wellbeing of those caring for family members at the end of their life
Source: BMC Palliat Care. 2023 Jul 20;22:98. doi: 10.1186/s12904-023-01225-1 (PMC10357776; doi:10.1186/s12904-023-01225-1)
Supplement: Supplementary file 3 — Additional File 3. The Australian Carer Toolkit for Advanced Disease: Focus Groups and Interviews. [file 12904_2023_1225_MOESM3_ESM.pdf]

Summary Report

The Australian Carer Toolkit for Advanced Disease: Scoping Study

March 2019

Prepared by:

Kristina Thomas

Dianne Saward

Peter Hudson

Summary Report - Scoping Study

Aims of Scoping Study:

1. To identify high quality existing web-based content and resources that focus on providing information to support Australian family carers of people with advanced disease.
2. To use identified web-based resources in the Carer Toolkit either as content (with permission) or as links.
3. To avoid duplication of effort by utilizing existing high quality material; where available.

Process: Step 1 - Identifying websites/online resources

Inclusion criteria: Websites that provided information to family carers of adult patients who have been diagnosed with an advanced life limiting disease such as cancer, dementia, neurological disease, heart/vascular disease, respiratory disease, motor neurone disease/ALS, kidney disease or liver disease.

The main focus was on Australian websites however seminal sites from UK, USA, Canada, or New Zealand were also included.

A website could include a number of individual pages or resources that were relevant to family carers of patients with advanced disease such as:

- information
- videos
- booklets or fact sheets
- interactive educational modules
- a page of links to other relevant organisations

The process for identifying websites incorporated:

A. Inviting each National Carer Toolkit Reference Group (NRG) member to contribute to the scoping study by identifying up to 4 websites and assessing the quality of these websites/online resources.
B. For those NRG members who did not respond to our request, we (the Carer Toolkit Management Committee) reviewed websites that were directly related to the organisation and constituents the NRG member represents.
C. The Executive Committee then brainstormed a list of websites that were also reviewed for the scoping study.
D. Websites of key international organisations (for example, The National Council for Palliative Care (UK))

The list of websites reviewed is listed in Appendix A.

Process: Step 2 – Review and assessment of websites / online resources

The Carer Toolkit Project Management Committee developed a Website Quality Assessment Form (refer to Appendix B) to use to review the websites in a systematic way. This form was approved by the Executive Committee. Each website was then reviewed using the Website Quality Assessment Form by either a NRG member or a member of the Carer Toolkit Management Committee.

This information from the Website Quality Assessment Forms for 50 websites was collected and compiled into a spreadsheet. The Carer Toolkit Management Committee reviewed the information and selected websites were useful for the Carer Toolkit based on:

1. The relevance of the information (it meets a 'need' for family caregivers of advanced disease)
2. The recency of the information (has it been updated in the last 5 years?)
3. Whether the content was evidence-based or from an organisation that had an evidenced-based philosophy (e.g. The Cancer Council).

Seventeen websites were excluded primarily because the information was patient specific or related to carers but in relation to survivorship.

Results

In total, 33 websites (see Table S 1) were included in the scoping study. Many of these websites included multiple pages and multiple resources that were relevant. Of those in scope, 4 were cancer related, 6 are disease specific (non-cancer) and 23 are generic (not related to any specific disease). The content areas of these 33 websites were presented at the second NRG meeting.

The key findings included:

1. There is already a lot of information on the majority of topic areas across a variety of websites. However, the difficulty for carers is finding the information in one place.
2. The disease specific sites, do not include much information on choosing site of care (e.g. home, hospital, aged care facility), however this is covered well by palliative care sites. If you are caring for someone who is not linked in with palliative care, then you would be unlikely to access this information.
3. The coping with emotions and difficult conversations content area is not frequently covered and this may be a priority area for our population. Similarly, changing roles and relationships is not frequently covered by disease specific sites.
4. There is a general lack of information that is specific to CALD populations, LGBTIQ populations or ATSI populations.
5. While there are videos around, they are of varying quality and content.
6. While death and dying is covered by many sites (generic and disease-specific), the information provided is more of an overview rather than detailed information. More detailed information on death and dying from a carers' perspective is a current gap.

Recommendations for the Carer Toolkit Project

The implications of this scoping study are that there is a need for a generic online resource for carers of people with advanced disease, particularly if they are not already linked into palliative care. There should be a focus on high quality information and links to other sites (where appropriate). There needs to be a focus on location of care, dealing with emotions and difficult conversations, changing roles and relationships and death and dying. The lack of detailed information on death and dying highlights a specific gap in current online resources that should be addressed in the Carer Toolkit project. Consideration to how the online resources are inclusive and relevant to CALD ATSI, and LGBTI populations should also be a priority.

Recommendations for Palliative Care

There is good quality palliative care information on palliative care and cancer websites which meets many of the needs of carers of people living with advanced disease. For those who are caring for someone with an advanced non-malignant condition, there is less content available to assist them with their caring role and preparation for death and dying. Location of care, at the end of life, is also missing from cancer specific and non-malignant specific online resources (except dementia) and dealing with emotions and difficult conversations is also not well explored across most sites. We recommend that palliative care has a role to promote itself and share its expertise through disease specific sites, especially content around the benefits of palliative care to carers of people living with an advanced disease, how to access palliative care, and preparedness for death and dying. Many people will die without specialist palliative care services, and therefore sharing palliative cares' expertise about death and dying to disease specific organisations would be a way of reaching a different audience.

Table S1: Results

Cancer Specific

Organisation	Year	Practical Care tasks	Negotiating the health system	Self care strategies	Location of care	Palliative Care as an option	Coping with emotions and difficult conversations	Changing roles and relationships	Videos re experience	Legal/ Financial	Care as death approaches	Death and dying	Grief and bereavement	CALD
Cancer Council Australia	2017	y	y	y		y	y	y	y	y	y	y	y	y
Prostate Cancer Foundation	2014			y										
Breast Cancer Network		y							y					
Queens University Belfast – Cancer Caring Coping		y		y					y				y	

Disease Specific (non-cancer)

Organisation	Year	Practical Care tasks	Negotiating the health system	Self care strategies	Location of care	Palliative Care as an option	Coping with emotions and difficult conversations	Changing roles and relationships	Videos re experience	Legal/Financial	Care as death approaches	Death and dying	Grief and bereavement	CALD
MND Australia	2018										y			
Dementia Australia	2017	y	y	y	y			y	y		y	y	y	y
MS Australia	2017	y		y						y				
Parkinson's Australia	2017	y		y		y	y	y			y	y		
The Stroke Foundation	2018	y		y					y					
Lung Foundation	2013					y			y	y	y			y

Generic

Organisation	Year	Practical Care tasks	Negotiating the health system	Self-care strategies	Location of care	Palliative Care as an option	Coping with emotions and difficult conversations	Changing roles and relationships	Videos re experience	Legal/Finan cial	Care as death approaches	Death and dying	Grief and bereavement	CALD
Grampians Pall Care	2016	y							y		y	y		
Eastern Palliative Care	2015	y							y					y
Loddon Mallee Palliative Care	2015	y												
Carer Gateway	2017	y			y	y		y	y	y	y	y	y	Y
Palliative Care Victoria		y		y	y	y			y			y	y	Y
Palliative Care Australia		y				y		y	y		y	y	y	Y
COTA Victoria	2018									y				
CareSearch	2017	y		y		y	y	y	y	y	y	y	y	Y
Health Direct		y	y											Y
Carers NSW	2010	y	y	y	y			y		y				Y
AIIHPC	2017	y		y	y	y	y		y	y	y	y	y	
Centre for Palliative Care	2018								y					
Carers Australia	2018	y	y	y	y	y		y			y	y	y	
Chronic Illness Alliance	2018		y							y				
Australia Centre Grief Bereavement	2018												y	
Centrelink	2018									y				Y
Better Health Channel	2015		y					y						Y
Hospice UK	2018	y		y	y	y			y					
Carers UK	2014	y			y		y	y		y				
Carers NZ	2018	y		y			y	y	y		y	y	y	Y
Dying Matters	2018										y	y	y	
ACP Australia	2018									y				Y

Appendix A. List of websites reviewed for scoping study

Australian Cancer Specific

- Cancer Australia
- Breast Cancer Network
- Council Cancer Australia
- Carers Couch
- Prostate Cancer Foundation
- Barwon Health
- Peter MacCallum Cancer Centre
- Leukaemia Foundation

Australian Disease Specific

- MND (Motor Neurone Disease)
- Dementia Australia
- MS (Multiple Sclerosis) Australia
- Lung Foundation
- Neurological Alliance Australia
- Parkinson's Australia
- Stroke Foundation
- HIV/AIDS National Federation of AIDS Organisation

Australian Generic

- COTA (Council of the Aging)
- Carer Gateway
- CareSearch
- Flinders University
- Health Direct
- Centre Link
- Carers Australia
- Carers NSW
- Chronic Illness Alliance
- Australian Centre for Grief and Bereavement
- FECCA (Federation of Ethnic Communities' Councils of Australia)
- Better Health Channel
- Dying Matters
- ACP (Advance Care Planning) Australia

Australian Palliative Care

- PalliAged
- Palliative Care Australia
- Centre for Palliative care
- Palliative Care Victoria

- Lodden Mallee Palliative Care Consortium
- Eastern Palliative Care
- Grampians Regional Palliative care
- Caring@home

International Cancer Specific

- Queens University Belfast

International Generic

- Carers UK
- Canadian Carers Coalition
- Family Caregiver Alliance (USA)
- Care Alliance Ireland
- National Alliance for Caregivers (USA)
- Carers Canada
- Eurocarers
- Carers NZ

International Palliative Care

- AIIHPC (All Ireland Institute of Hospice and Palliative Care)
- Hospice UK
- The National Council for Palliative Care UK

Appendix B. Website Quality Assessment Form

Note: Please complete 1 form for each website /online resource (up to 4 per NRG member).

Name of person completing form: ________________________ **Date:** ____________________

1. Website organisation/name:

2. Website link/s:

   Please provide details of specific web pages or resources that are on this website that are relevant to family carers with advanced disease.

3. A) Who is the target audience for resource?

   B) Is it disease specific or generic enough to be relevant for all family carers?

4. What information needs does the resource meet?

5. How well does it meet the need? How could we improve it?

6. How was the resource developed?

7. How recent is the resource? When was the resource last updated?

8. Is the resource located on the website of a peak body?

9. Is the resource promoted by an organisation that claims evidence-based practice?

10. Are you aware of any evaluation on the effectiveness/ usefulness of the resource? Please describe or provide a link.

11. Do you think we should seek to include this content/resource in our toolkit (with permission) or do you think our toolkit should just link to that section or the whole site?

12. Any other comments?
