# Supplementary material for: Who cares for the carers? carerhelp: development and evaluation of an online resource to support the wellbeing of those caring for family members at the end of their life
Source: BMC Palliat Care. 2023 Jul 20;22:98. doi: 10.1186/s12904-023-01225-1 (PMC10357776; doi:10.1186/s12904-023-01225-1)
Supplement: Supplementary file 4 — Additional File 4: CarerHelp Website User Testing Review Form. [file 12904_2023_1225_MOESM4_ESM.pdf]

The Australian Carer Toolkit for Advanced Disease: Literature Review

REPORT PREPARED FOR THE NATIONAL REFERENCE GROUP

PREPARED BY

DAVID MARCO

KRISTINA THOMAS

PETER HUDSON

THE CENTRE FOR PALLIATIVE CARE

ST VINCENT'S HOSPITAL MELBOURNE & THE UNIVERSITY OF MELBOURNE

February, 2019

Contents

Executive summary 3

Literature review 3

Key findings....3

Recommendations 3

Methods.... 4

Inclusion and exclusion criteria 4

Searches and information sources.... 4

Study selection and data extraction 4

Data analysis and synthesis 4

Results 6

Conclusions 7

Limitations 8

Recommendations 8

Acknowledgements.... 8

Glossary 10

References 12

Executive summary

Many family carers of people with advanced disease report unmet needs in information provision and support. In 2017, the Centre for Palliative Care, in collaboration with a range of national organisations, was commissioned by the Federal Government to commence work on the *Australian Carer Toolkit for Advanced Disease* ("Carer Toolkit"). This project aims to develop a centralised online resource for family carers seeking information and support to assist them in their caregiving role.

Literature review

As part of the project, the Centre for Palliative Care commenced a systematically-conducted literature review of reviews. The purpose of this review was to identify the core needs of family carers of people with advanced disease. The results from this report will inform the next phase of the project; developing the content for the online Carer Toolkit.

Key findings

Results from the database search identified 2674 potentially relevant articles, of which 68 met the inclusion criteria for this review. Information regarding reported carer needs and disease cohort was extracted from each article and illustrated for the specific purposes of this report.

Key findings were:

- A wide range of carer needs were reported across diseases. Needs were largely representative of four main categories: *Communication*, *Education*, *Practical*, and *Psychosocial* support. The most commonly reported needs were from the *Education* category.
- Disease cohorts were from the following main categories: *Advanced disease (non-specific)*, *Malignant*, and *Non-malignant*. The majority of papers investigated carer needs in the *Advanced disease (non-specific)* category.
- The single most highly reported need across all diseases was "practical support" with respect to the role of the carer. The top four reported needs ("symptom management", "carer role", and education about "prognosis" and "diagnosis") were documented most frequently in the "cancer" and "end-of-life" disease cohorts.
- Carers looking after people from motor neuron disease, chronic obstructive pulmonary disease, cancer, palliative care, glioma, and end-of-life disease cohorts reported the widest range of needs spanning across all four need categories.

Recommendations

The following recommendations were made in response to these findings:

- The Carer Toolkit should address unmet needs in domains relating to *Communication*, *Education*, *Practical*, and *Psychosocial* support.
- Despite the prevalence of some disease-specific needs, most reported needs were universal across the disease spectrum. Therefore, the Carer Toolkit resource would benefit from delivering information in a role-specific focus, rather than disease-specific. Suggested main topics could include:
    - *What should I expect?* (e.g. education about disease and prognosis)
    - *What is expected of me?* (e.g. the role of the carer)
    - *How will I be affected?* (e.g. psychosocial implications)
    - *Who can help me?* (e.g. resources and support available)

The results in this report will be subject to further ongoing investigation, which will inform a subsequent systematic review paper to be submitted to a peer-reviewed journal for public dissemination.

The purpose of this report is to outline methods, findings and key recommendations resulting from a systematically-conducted literature review of reviews aimed at investigating the core needs of family carers of people with advanced disease.

Methods

The overarching methodology used was a 'systematic review of reviews' approach[1]. In the health literature, decision makers are increasingly faced by a plethora of systematic reviews, likely to be of variable quality and scope. Systematic reviews of reviews allow findings from separate reviews to be compared and contrasted, bringing together a summary of reviews in one place to aid evidence-based clinical decision-making.

A senior research librarian at St Vincent's Hospital Melbourne medical library, experienced in systematic reviews and family carer literature, assisted with development of the initial search strategy, article extraction, and drafting of literature review methodology.

Inclusion and exclusion criteria

Papers were included if they met the eligibility criteria reported in Table s1. All articles reporting a 'need' were included. The definition of 'need' was operationalised by the following rules:

1. A resource identified by the carer that is desired but currently lacking
2. Is not an 'experience' or 'lack of experience' or 'burden' or a 'challenge'
3. Papers where "carer needs" were referred to but specific needs were not identified were rejected (e.g. "carers put patient needs ahead of their own" or "carers needs are often overlooked")

Searches and information sources

Searches were conducted in the following bibliographic databases:

- MEDLINE
- Emcare
- Embase
- PsycINFO
- CINAHL
- Cochrane Library
- Informit

Searches were completed by December 2017 and identified papers published between 1 January 2007 and 30 November 2017. Refer to the Appendix for full details of the search strategy.

Study selection and data extraction

All titles and abstracts were screened independently by DM and KT. Screening of full-text articles was undertaken by DM with assistance from KT. Disagreements about eligibility for inclusion were resolved by discussion, or by referral to PH.

Data analysis and synthesis

For the purposes of this report, data for analysis comprised tallying the needs of carers reported in

Table s1 *Inclusion/exclusion criteria*

Inclusion criteria	Exclusion Criteria
- Review articles:<br>- systematic reviews<br>- meta-analyses<br>- systematically-conducted reviews<br>- critical reviews<br>- meta ethnography<br>- meta/narrative synthesis<br>- Last 10 years; Jan 2007 – Nov 2017<br>- The investigation of carer needs is reported in the aims and results section of the review<br>- English language articles	- Carer satisfaction with services<br>- Formal/paid carers<br>- Carer's role with respect to the needs of others<br>- Intervention papers not identifying carer needs<br>- Health economics papers<br>- Paediatric patients/carers<br>- Animal studies<br>- Protocol papers<br>- Literature reviews in papers with original research<br>- Literature reviews of scales, tools, measures, and quality indicators<br>- Book reviews

Table s2 List of disease cohorts and reported needs

Disease cohort	Label	Citations
Advanced disease (non-specific)		
End-of-life	EoL	[2-15]
Palliative care	PC	[16-24]
Malignant		
Cancer (general)	Cancer	[25-36]
Gliomas	Glioma	[37-40]
Haematological	Haem	[41]
Ovarian	Ovarian	[42]
Prostate	Prostate	[43]
Non-malignant		
Alzheimer's	Alzheimers	[44]
Breathlessness	Breath	[45, 46]
Chronic kidney disease	CKD	[47]
Chronic obstructive pulmonary disease	COPD	[48-54]
Delirium	Delirium	[55]
Dementia	Dementia	[56-58]
Diabetes	Diabetes	[59]
Heat failure	HF	[60-63]
Motor nurone disease	MND	[64-68]
Stroke	Stroke	[69]

Needs*	Label	Citations
Communication		
Family/patient	Family	[3, 18, 23-25, 33, 37, 42, 44, 51-53]
Health professionals	HP	[3, 8, 9, 13, 14, 16, 18, 23, 24, 33, 37-39, 41, 42, 53, 59, 60, 64, 67]
Contact with professional	Professional	[3, 8, 10, 11, 26, 33, 38, 39, 44, 45, 48, 49, 54, 58, 61, 65-68]
Education		
Diagnosis	Diagnosis	[3-5, 7, 10, 12, 15, 16, 18, 23, 27, 28, 30, 32, 33, 36, 38, 43, 45, 49-51, 53, 55, 61, 64, 68]
Prognosis	Prognosis	[3, 6, 11, 12, 15, 16, 23, 27, 28, 30, 32-34, 38, 42, 51-55, 59, 62, 64, 67, 69]
Symptom management	Symptoms	[6, 11-13, 16, 17, 21, 26, 27, 30, 32, 33, 35, 39, 41, 45, 50, 52, 55, 59, 62, 64, 68]
Treatment/medication	Treatment	[3, 5, 7, 11, 12, 16, 23, 26, 33, 35, 38, 42, 43, 45, 53-55, 59, 64]
Practical		
Coping	Coping	[2, 6, 12, 18, 33, 38, 45, 51, 60, 62, 68]
Emergency	Emergency	[12, 16, 26, 48, 54, 62]
Equipment	Equipment	[23, 26, 58, 66, 68, 69]
Finance	Finance	[14, 16, 23, 31, 38, 61, 62, 64, 66, 68]
Home	Home	[14, 23, 30, 32, 45, 46, 63, 64, 66, 68]
Resources	Resources	[8, 11-13, 15, 16, 21, 26, 28, 31, 33, 43, 51, 62, 68, 69]
Respite	Respite	[11, 12, 14, 23, 30, 39, 41, 49, 64, 66, 67]
Role	Role	[4, 8, 9, 11, 14-17, 20, 23, 28, 32, 33, 35, 36, 38, 39, 41, 44-46, 54, 56, 59, 61, 65, 67-69]
Psychosocial		
Acknowledgement	Acknowledge	[10, 13, 17, 22, 68]
Bereavement	Bereave	[2, 10, 12, 19, 23, 24, 27, 33, 38, 40, 54, 58]
Emotional	Emotional	[3, 10, 11, 15, 18, 23, 27, 32, 33, 35, 39, 40, 45, 47, 49, 50, 54, 56, 61, 62, 67-69]
Self	Self	[11, 16, 23, 26, 33, 41, 43, 51, 68, 69]
Social	Social	[3, 8, 11, 18, 19, 23, 32, 38, 42, 50, 57, 61-63, 66, 68, 69]
Spiritual	Spiritual	[3, 6, 10, 11, 15, 18, 23, 29, 32, 33, 61, 62]

Note: The label column represents the terminology used in subsequent illustrations (Figs S2-4).
*Refer to glossary for definition of reported need labels.

the results and discussion sections of each included paper along with the disease cohort being cared for. Results were classified into broader need and disease categories, where possible, and analysed descriptively using frequency histograms and heatmap matrices.

Results

The review process is shown in Figure s1. Of the 4483 possible articles identified, 2674 remained following removal of duplicates. Title and abstract screening identified 255 potentially eligible papers, and full-paper reading led to 68 reviews that met the criteria for inclusion.

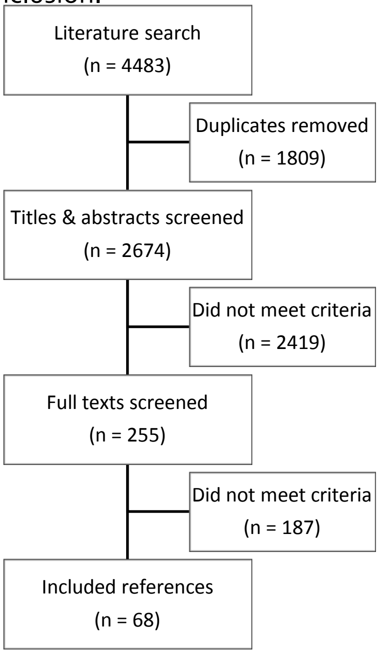

Figure s1 *Review process*

Table s2 shows the number of articles specific to each disease cohort. Diseases were grouped into three categories: *advanced disease (non-specific)*, *malignant*, and *non-malignant*. It must be noted that papers referring to "end-of-life" and "palliative care" cohorts could comprise individuals from the other disease categories. Irrespective of the method that authors stated that they had employed, the body of reviews were largely descriptive in their approach to data analysis.

Most reviews focused on carer reported needs in the areas of *communication*, *education*, *practical* support, and *psychosocial* support (see Table s2). Figure s2 illustrates the proportion of needs reported for each disease cohort adjusted for the number of papers reviewed.

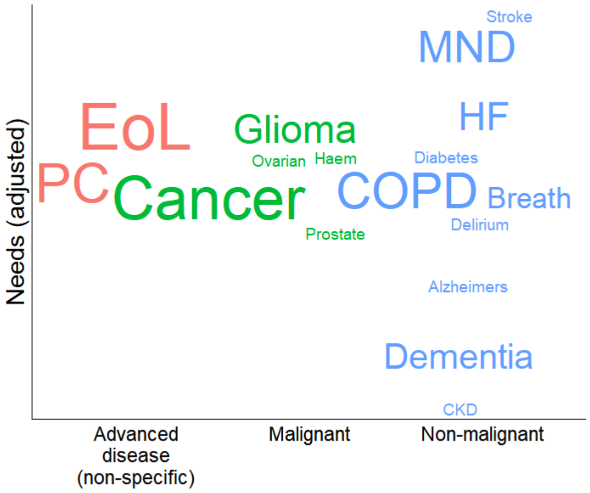

Figure s2 *Word chart illustrating the number of articles and the proportion of needs reported for each disease cohort. The size of the text in the chart represents the magnitude of articles investigating the disease cohort (smaller text = fewer articles; larger text = more articles). The vertical placement of the text illustrates the overall proportion of needs reported for that disease cohort as a function of the number of articles (higher text = more needs reported; lower text = fewer needs reported).*

The illustration suggests that while most articles investigated carer needs in the more general *advanced disease* and *cancer* groups, a proportionally higher number of needs were evident in the stroke, motor neurone disease, heart failure and glioma disease cohorts. Contrary to this, the fewest reported carer needs were found for chronic kidney disease, dementia, and Alzheimer's disease cohorts.

Figure S3 extrapolates the reported needs, grouped into the four main need categories identified in Table s2. The most commonly reported needs were from the *Education* category. However, the single most highly reported need across all disease cohorts was "role" (see Glossary for definitions of reported need labels).

Figure S4 illustrates a cross-tabulation of reported carer needs across all disease cohorts identified in this review. The illustration shows that the most

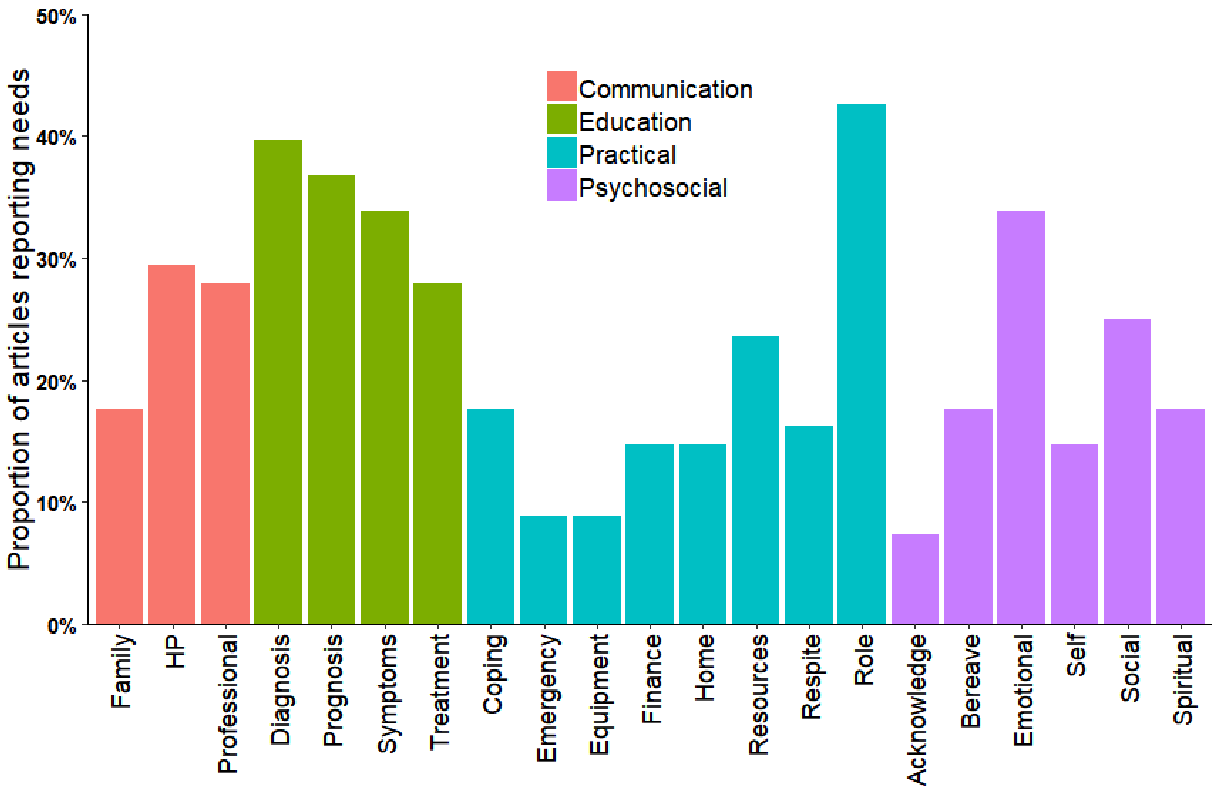

Figure S3 *Frequency histogram of reported needs grouped into four main categories. See Glossary for definitions of axis labels.*

frequently reported needs were in the "end-of-life" and "cancer" disease groups. However, carers looking after people from the motor neuron disease, chronic obstructive pulmonary disease, cancer, palliative care, glioma, and end-of-life disease cohorts reported the widest range of needs, spanning across all four need categories.

Conclusions

This review aimed to identify the core needs reported by family carers of people with advanced diseases for the purposes of informing the development of an online resource tool.

Results identified 68 articles which met the criteria for this review, encompassing diseases across both malignant and non-malignant cohorts. Twenty-one different needs were reported spanning four main categories: *communication*, *education*, *practical*, and *psychosocial* support.

Analyses suggest that carers from most disease cohorts had similar proportions of needs (Figure S2). However, on average, most needs came from the *education* category, indicating that carers were uncertain about information regarding the nature of the disease, its prognosis, treatment options, and symptom management. Coincidentally, this could explain why "role" was the most frequently reported need overall (Figure S3).

No obvious patterns emerged to suggest the prevalence of disease-specific needs (Figure S4). Instead, the most frequently reported needs were found in the "end-of-life" and "cancer" disease cohorts (i.e. non-specific disease groups). Most disease-specific cohorts had small numbers of reported needs, meaning that we could not confidently identify pertinent needs for those groups. The motor neurone disease, chronic obstructive pulmonary disease, palliative care, glioma, end-of-life, and cancer disease cohorts showed a similar spread of reported needs across all categories suggesting that carers from these groups could potentially benefit the most from the *Carer Toolkit* resource.

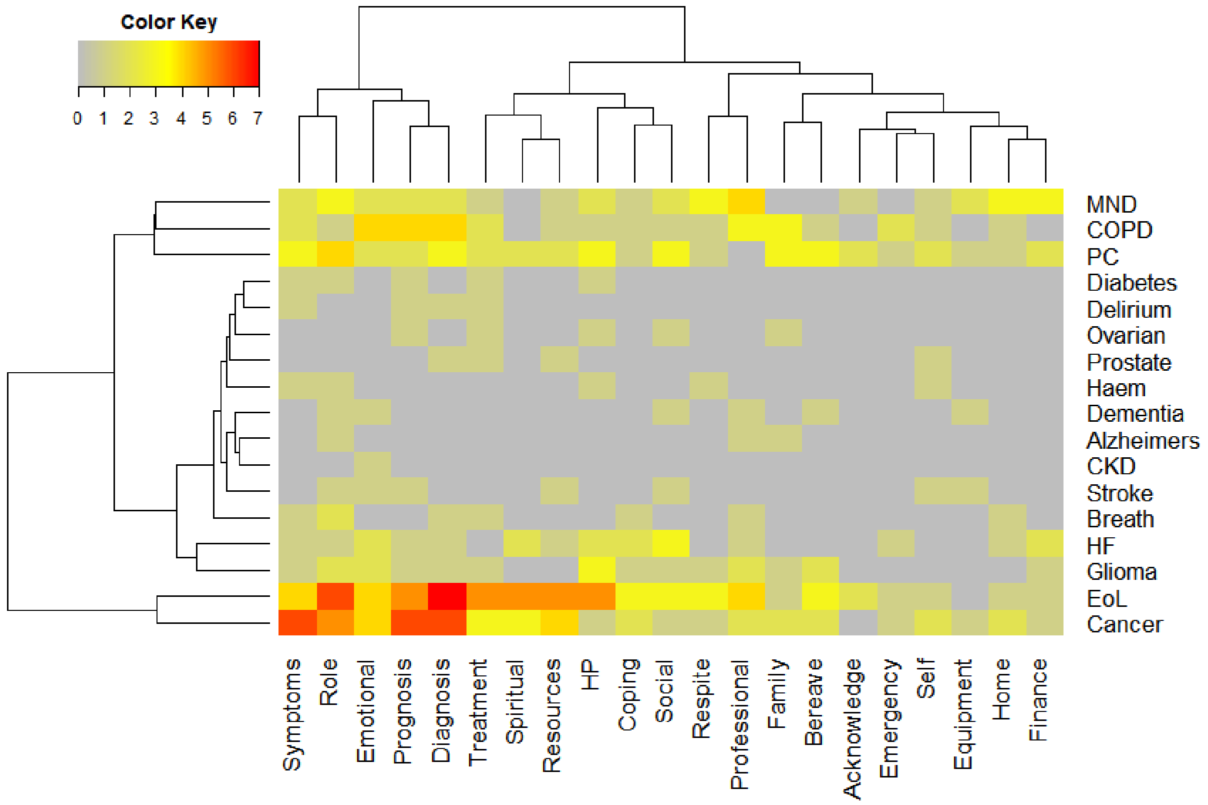

Figure S4 *Heatmap illustrating reported needs across disease cohorts*

Based on the needs reported in this review, we propose that carers are seeking help with the following questions:

- *What should I expect when looking after someone with advanced disease?*
- *What is expected of me as a carer?*
- *How will my life be affected?*
- *Where can I find help?*

These questions draw upon elements spanning the four needs categories identified in this review. They are reflective of the need for preparedness and resolving uncertainty. Addressing these elements should be the focus of the next phase of the development of the *Carer Toolkit* resource.

Limitations

This review had a few limitations which should be considered when interpreting these results. Firstly, not all needs reported were attributed to a specific disease. For example, articles investigating "end-of-life" and "palliative care" cohorts would presumably comprise a range of malignant and non-malignant conditions. For this reason, caution needs to be exercised when describing the needs of carers for a specific disease groups.

Secondly, the needs reported in a defined cohort cannot be considered exhaustive. In many articles, additional constraints were placed in the aims of the reviews (e.g. "*spiritual* needs when caring for cancer patients", or "the needs of *rural* carers of people with dementia", etc.).

Finally, for the purposes of this report, a formal assessment research quality was not conducted due to resource limitations. It could, therefore, be reasonably assumed that different weights would be applied, based on research merit, to information presented from each paper in this review.

Recommendations for the Carer Toolkit Project

The *Carer Toolkit* project should consider the following recommendations in the next phase of development:

- The Carer Toolkit should address unmet needs in domains relating to *Communication*, *Education*, *Practical*, and *Psychosocial* support.
- Despite the prevalence of some disease-specific needs, most reported needs were universal across the disease spectrum. Therefore, the Carer Toolkit resource would benefit from delivering information in a role-specific focus, rather than disease-specific. Suggested main topics could include:
    - *What should I expect?* (e.g. education about disease and prognosis)
    - *What is expected of me?* (e.g. the role of the carer)
    - *How will I be affected?* (e.g. psychosocial implications)
    - *Who can help me?* (e.g. resources and support available)

The results in this report will be subject to further investigation, which will inform a subsequent systematic review paper to be submitted to a peer-reviewed journal for public dissemination.

Implications for Palliative Care

Carers of people living with an advanced disease have many unmet needs that fall within the remit of palliative care. Palliative care services should offer comprehensive support to carers including: education on symptoms and prognosis, preparation for the role of becoming a carer; support with psychosocial concerns during caring and bereavement; and support and opportunity to effectively communicate with the health care team and other family members.

Acknowledgements

- Ms Helen Wilding (Senior Research Librarian, Centre for Palliative Care)
- Prof. Jennifer Tieman (Director, CareSearch and Dean, Research of the College of Nursing and Health Sciences, Flinders University)
- Prof. Deborah Parker (Professor of Nursing Aged Care, University of Technology Sydney)

Glossary

Acknowledgement wish to be formally acknowledged as the caregiver and duties the carer is responsible for

Bereavement support at/after the dying and death stages

Contact with professional direct access to a health professional or assigned case manager when required

Coping support (practical and emotional) for coping with the caregiver role; balancing with other priorities; managing daily responsibilities

Diagnosis information/education regarding the nature of the illness/disease/diagnosis

Emergency a direct point of contact for the carer when an emergency arises (e.g. symptom management, rapid deterioration, unexpected events, existential crisis, etc.)

Emotional support with psychosocial challenges experienced by carer or patient

Equipment apparatus/tools/physical aids to assist with care and transport of the patient

Finance support with finances and financial advice

Health professionals improvements/facilitation/clarity in communication between health professional and the carer and/or patient

Home assistance with regular household tasks (distinct from carer duties)

Family assistance with facilitating communication between family members and/or patient

Prognosis information/education regarding the likely course/outcome of the disease

Respite opportunity for respite

Resources being informed of, and having access to, available resources for carers and patients

Role education/support/guidance in being a carer with respect to responsibilities, duties, and expectations

Self support looking after personal self (carer)

Social support networks and relationships with others outside the caring role

Spiritual faith-based support and guidance

Symptom management information/education regarding the management of disease symptoms and treatment side-effects

Treatment/medication information/education regarding treatment and/or medication the patient is receiving

Appendix

MEDLINE search strategy

1. caregivers/ or family/ or exp family relations/ or spouses/ or siblings/ or parents/ or fathers/ or mothers/ or grandparents/ or adult children/ or friends/ or sexual partners/ or exp legal guardians/
2. (caregiv* or care giver* or care giving or carer* or family or families or spous* or wife or wives or husband* or parent or parents or father* or mother* or sibling* or brother* or sister* or partner* or adult child* or friend* or neighbour* or loved one or relative*).ti.
3. 1 or 2
4. palliative care/ or terminal care/ or hospice care/ or hospices/ or "hospice and palliative care nursing"/ or palliative medicine/ or terminally ill/
5. attitude to death/ or advance care planning/ or advance directives/
6. (palliat* or hospice* or terminal* or end of life or dying or life limit* or life threatening or incurable or end stage).ti,ab.
7. (critically ill or critical illness or advance care plan* or advanced care plan* or advance directive* or advanced directive* or late stage care).ti,ab.
8. *motor neuron disease/ or *amyotrophic lateral sclerosis/ or *pulmonary disease, chronic obstructive/ or *renal insufficiency, chronic/ or *kidney failure, chronic/
9. (motor neurone or amyotrophic lateral sclerosis or ALS or COPD or chronic obstructive pulmonary disease or chronic renal insufficiency or chronic renal failure or chronic kidney failure).ti.
10. (high grade or stage 4 or metastatic or advanced or refractory or severe or progressive or end or late).ti.
11. exp *neoplasms/ or *Parkinson disease/ or *Huntington disease/ or *stroke/ or exp *dementia/ or *heart failure/ or exp *Kidney Diseases/ or exp *Multiple Sclerosis/
12. (disease* or illness or cardiovascular or neurological or degenerative or kidney or renal or liver or heart or stroke or dementia or alzheimer* or multiple sclerosis or cancer* or tumor* or tumour* or glioma* or leukaemia or leukemia or lymphoma or mesothelioma or melanoma or myeloma).ti.
13. 11 or 12
14. 10 and 13
15. 4 or 5 or 6 or 7 or 8 or 9 or 14
16. "review"/ or meta-analysis/
17. (review or meta analy* or metaanaly* or meta ethnograph* or metaethnograph* or metasynthesis or meta synthesis or narrative synthesis).ti,ab.
18. (literature search* or prisma or medline or pubmed or cinahl or qualitative literature).ti,ab.
19. 16 or 17 or 18
20. 3 and 15 and 19
21. animals/ or (pediat* or paediat* or perinatal or stillbirth or stillborn or neonat* or infant* or newborn).ti.
22. 20 not 21
23. limit 22 to (english language and yr="2007 -Current")

References

1. Smith, V., et al., Methodology in conducting a systematic review of systematic reviews of healthcare interventions. BMC Med Res Methodol, 2011. **11**(1): p. 15.

2. Broady, T.R., Carers' Experiences of End-of-Life Care: A Scoping Review and Application of Personal Construct Psychology. Australian Psychologist, 2017. **52**(5): p. 372-380.

3. Chan, M.M.Y. and S.Y. Chair, The needs of family members of critically ill patients: a literature review. CONNECT: The World of Critical Care Nursing, 2008. **6**(2): p. 30-33.

4. Funk, L., et al., Part 2: Home-based family caregiving at the end of life: a comprehensive review of published qualitative research (1998-2008). Palliative Medicine, 2010. **24**(6): p. 594-607.

5. Hancock, K., et al., Discrepant perceptions about end-of-life communication: A systematic review. Journal of Pain and Symptom Management, 2007. **34**(2): p. 190-200.

6. He Leow, M.Q. and S. Wai Chi Chan, Factors affecting caregiver burden of terminally ill adults in the home setting - A systematic review. JBI Library of Systematic Reviewis, 2011. **9**(45): p. 1883-1916.

7. Jo, M., et al., Family-Clinician Communication about End-of-Life Care in Korea: A Narrative Review. Journal of Hospice and Palliative Nursing, 2017. **19**(6): p. 597-601.

8. Kirby, S., et al., Are rural and remote patients, families and caregivers needs in life-limiting illness different from those of urban dwellers? A narrative synthesis of the evidence. Australian Journal of Rural Health, 2016. **24**(5): p. 289-299.

9. Lowey, S.E., Communication between the nurse and family caregiver in end-of-life care: A review of the literature. Journal of Hospice and Palliative Nursing, 2008. **10**(1): p. 35-48.

10. Melin-Johansson, C., et al., Living in the presence of death: an integrative literature review of relatives' important existential concerns when caring for a severely ill family member. The Open Nursing Journal, 2012. **6**: p. 1-12.

11. Morris, S.M., et al., Family carers providing support to a person dying in the home setting: A narrative literature review. Palliative Medicine, 2015. **29**(6): p. 487-95.

12. Parker, S.M., et al., A systematic review of prognostic/end-of-life communication with adults in the advanced stages of a life-limiting illness: patient/caregiver preferences for the content, style, and timing of information. Journal of Pain & Symptom Management, 2007. **34**(1): p. 81-93.

13. Rainsford, S., et al., Rural end-of-life care from the experiences and perspectives of patients and family caregivers: A systematic literature review. Palliative Medicine, 2017. **31**(10): p. 895-912.

14. Stajduhar, K., et al., Part 1: Home-based family caregiving at the end of life: a comprehensive review of published quantitative research (1998-2008). Palliative Medicine, 2010. **24**(6): p. 573-93.

15. Yoo, J.S., J. Lee, and S.J. Chang, Family Experiences in End-of-Life Care: A Literature Review. Asian Nursing Research, 2008. **2**(4): p. 223-34.

16. Arias Rojas, M. and C. Garcia-Vivar, The transition of palliative care from the hospital to the home: a narrative review of experiences of patients and family caretakers. Investigacion y Educacion en Enfermeria, 2015. **33**(3): p. 482-491.

17. Chi, N.C. and G. Demiris, Family Caregivers' Pain Management in End-of-Life Care: A Systematic Review. American Journal of Hospice & Palliative Medicine, 2017. **34**(5): p. 470-485.

18. Docherty, A., et al., Knowledge and information needs of informal caregivers in palliative care: a qualitative systematic review. Palliative Medicine, 2008. **22**(2): p. 153-71.

19. Holtslander, L., et al., Honoring the voices of bereaved caregivers: a Metasummary of qualitative research. BMC Palliative Care, 2017. **16**(1): p. 48.

20. Hudson, P. and S. Payne, Family caregivers and palliative care: current status and agenda for the future. Journal of Palliative Medicine, 2011. **14**(7): p. 864-9.

21. Robinson, C.A., et al., Rural palliative care: A comprehensive review. Journal of Palliative Medicine, 2009. **12**(3): p. 253-258.

22. Veloso, V.I. and V.A. Tripodoro, Caregivers burden in palliative care patients: a problem to tackle. Current Opinion in Supportive & Palliative Care, 2016. **10**(4): p. 330-335.

23. Ventura, A.D., et al., Home-based palliative care: a systematic literature review of the self-reported unmet needs of patients and carers. Palliative Medicine, 2014. **28**(5): p. 391-402.

24. Zaider, T. and D. Kissane, The assessment and management of family distress during palliative care. Current Opinion in Supportive & Palliative Care, 2009. **3**(1): p. 67-71.

25. Aamotsmo, T. and K.E. Bugge, Balance artistry: the healthy parent's role in the family when the other parent is in the palliative phase of cancer--challenges and coping in parenting young children. Palliative & Supportive Care, 2014. **12**(4): p. 317-29.

26. Bee, P.E., P. Barnes, and K.A. Luker, A systematic review of informal caregivers' needs in providing home-based end-of-life care to people with cancer. Journal of Clinical Nursing, 2009. **18**(10): p. 1379-93.

27. Clark, K., Care at the very end-of-life: Dying cancer patients and their chosen family's needs. Cancers, 2017. **9**(2).

28. Duggleby, W., et al., A metasynthesis study of family caregivers' transition experiences caring for community-dwelling persons with advanced cancer at the end of life. Palliative Medicine, 2017. **31**(7): p. 602-616.

29. Ferrell, B.R. and P. Baird, Deriving Meaning and Faith in Caregiving. Seminars in Oncology Nursing, 2012. **28**(4): p. 256-261.

30. Glajchen, M., Physical well-being of oncology caregivers: an important quality-of-life domain. Seminars in Oncology Nursing, 2012. **28**(4): p. 226-35.

31. Hanratty, B., et al., Review article: Financial stress and strain associated with terminal cancer a review of the evidence. Palliative Medicine, 2007. **21**(7): p. 595-607.

32. Kim, Y. and C.S. Carver, Recognizing the value and needs of the caregiver in oncology. Current Opinion in Supportive & Palliative Care, 2012. **6**(2): p. 280-8.

33. Lambert, S.D., et al., The unmet needs of partners and caregivers of adults diagnosed with cancer: a systematic review. BMJ supportive & palliative care, 2012. **2**(3): p. 224-30.

34. Northfield, S. and M. Nebauer, The caregiving journey for family members of relatives with cancer: how do they cope? Clinical Journal of Oncology Nursing, 2010. **14**(5): p. 567-77.

35. Ugalde, A., M. Krishnasamy, and P. Schofield, Supporting informal caregivers of people with advanced cancer: a literature review. Australian Journal of Cancer Nursing, 2011. **12**(2): p. 12-16.

36. Wheelwright, S., et al., A systematic review and thematic synthesis of quality of life in the informal carers of cancer patients with cachexia. Palliative Medicine, 2016. **30**(2): p. 149-160.

37. Ford, E., et al., Systematic review of supportive care needs in patients with primary malignant brain tumors. Neuro-Oncology, 2012. **14**(4): p. 392-404.

38. Moore, G., et al., Palliative and supportive care needs of patients with high-grade glioma and their carers: a systematic review of qualitative literature. Patient Education & Counseling, 2013. **91**(2): p. 141-53.

39. Sterckx, W., et al., The impact of a high-grade glioma on everyday life: a systematic review from the patient's and caregiver's perspective. European Journal of Oncology Nursing, 2013. **17**(1): p. 107-17.

40. Willemijn Boele, F., R. Grant, and P. Sherwood, Challenges and support for family caregivers of glioma patients. British Journal of Neuroscience Nursing, 2017. **13**(1): p. 8-16.

41. Beattie, S. and S. Lebel, The experience of caregivers of hematological cancer patients undergoing a hematopoietic stem cell transplant: a comprehensive literature review. Psycho-Oncology, 2011. **20**(11): p. 1137-50.

42. Petricone-Westwood, D. and S. Lebel, Being a caregiver to patients with ovarian cancer: A scoping review of the literature. Gynecologic Oncology, 2016. **143**(1): p. 184-192.

43. Sinfield, P., et al., Men's and carers' experiences of care for prostate cancer: a narrative literature review. Health Expectations, 2009. **12**(3): p. 301-12.

44. Kettl, P., Helping families with end-of-life care in Alzheimer's disease. Journal of Clinical Psychiatry, 2007. **68**(3): p. 445-50.

45. Farquhar, M., Carers and breathlessness. Current Opinion in Supportive and Palliative Care, 2017. **11**(3): p. 165-173.

46. Janssen, D.J., E.F. Wouters, and M.A. Spruit, Psychosocial consequences of living with breathlessness due to advanced disease. Current Opinion in Supportive & Palliative Care, 2015. **9**(3): p. 232-7.

47. Walker, R.C., et al., Patient and caregiver perspectives on home hemodialysis: a systematic review. American Journal of Kidney Diseases, 2015. **65**(3): p. 451-63.

48. Boyle, A.H., An integrative review of the impact of COPD on families. Southern Online Journal of Nursing Research, 2009. **9**(3): p. 6p-6p.

49. Caress, A.L., et al., A review of the information and support needs of family carers of patients with chronic obstructive pulmonary disease. Journal of Clinical Nursing, 2009. **18**(4): p. 479-91.

50. Cruz, J., A. Marques, and D. Figueiredo, Impacts of COPD on family carers and supportive interventions: a narrative review. Health & Social Care in the Community, 2017. **25**(1): p. 11-25.

51. Farquhar, M., Assessing carer needs in chronic obstructive pulmonary disease. Chronic Respiratory Disease, 2017: p. 1479972317719086.

52. Grant, M., A. Cavanagh, and J. Yorke, The impact of caring for those with chronic obstructive pulmonary disease (COPD) on carers' psychological well-being: a narrative review. International Journal of Nursing Studies, 2012. **49**(11): p. 1459-71.

53. Mathews, G. and B. Johnston, Palliative and end-of-life care for adults with advanced chronic obstructive pulmonary disease: a rapid review focusing on patient and family caregiver perspectives. Current Opinion in Supportive & Palliative Care, 2017. **11**(4): p. 315-327.

54. Nakken, N., et al., Informal caregivers of patients with COPD: Home Sweet Home? European Respiratory Review, 2015. **24**(137): p. 498-504.

55. Finucane, A.M., et al., The experiences of caregivers of patients with delirium, and their role in its management in palliative care settings: an integrative literature review. Psycho-Oncology, 2017. **26**(3): p. 291-300.

56. Barker, S., M. Lynch, and J. Hopkinson, Decision making for people living with dementia by their carers at the end of life: a rapid scoping review. International Journal of Palliative Nursing, 2017. **23**(9): p. 446-456.

57. Hennings, J. and K. Froggatt, The experiences of family caregivers of people with advanced dementia living in nursing homes, with a specific focus on spouses: A narrative literature review. Dementia, 2016. **17**: p. 17.

58. Peacock, S.C., The experience of providing end-of-life care to a relative with advanced dementia: an integrative literature review. Palliative & Supportive Care, 2013. **11**(2): p. 155-68.

59. Dikkers, M.F., T. Dunning, and S. Savage, Information needs of family carers of people with diabetes at the end of life: a literature review. Journal of Palliative Medicine, 2013. **16**(12): p. 1617-23.

60. Cagle, J.G., et al., Psychosocial needs and interventions for heart failure patients and families receiving palliative care support: a systematic review. Heart Failure Reviews, 2017. **22**(5): p. 565-580.

61. Doherty, L.C., D. Fitzsimons, and S.J. McIlfatrick, Carers' needs in advanced heart failure: A systematic narrative review. European Journal of Cardiovascular Nursing, 2016. **15**(4): p. 203-12.

62. Nicholas Dionne-Odom, J., et al., Family caregiving for persons with heart failure at the intersection of heart failure and palliative care: a state-of-the-science review. Heart Failure Reviews, 2017. **22**(5): p. 543-557.

63. Whittingham, K., S. Barnes, and C. Gardiner, Tools to measure quality of life and carer burden in informal carers of heart failure patients: a narrative review. Palliative Medicine, 2013. **27**(7): p. 596-607.

64. Aoun, S.M., et al., A 10-year literature review of family caregiving for motor neurone disease: moving from caregiver burden studies to palliative care interventions. Palliative Medicine, 2013. **27**(5): p. 437-46.

65. Bergin, S. and C. Mockford, Recommendations to support informal carers of people living with motor neurone disease. British Journal of Community Nursing, 2016. **21**(10): p. 518-524.

66. Harris, M., et al., Supporting wellbeing in motor neurone disease for patients, carers, social networks, and health professionals: A scoping review and synthesis. Palliative & Supportive Care, 2017: p. 1-10.

67. Holkham, L. and A. Soundy, The experience of informal caregivers of patients with motor neurone disease: A thematic synthesis. Palliative & Supportive Care, 2017: p. 1-10.

68. Oh, J. and J.A. Kim, Supportive care needs of patients with amyotrophic lateral sclerosis/motor neuron disease and their caregivers: A scoping review. Journal of Clinical Nursing, 2017. **05**: p. 05.

69. Creutzfeldt, C.J., R.G. Holloway, and M. Walker, Symptomatic and palliative care for stroke survivors. Journal of General Internal Medicine, 2012. **27**(7): p. 853-60.
