# Supplementary material for: Who cares for the carers? carerhelp: development and evaluation of an online resource to support the wellbeing of those caring for family members at the end of their life
Source: BMC Palliat Care. 2023 Jul 20;22:98. doi: 10.1186/s12904-023-01225-1 (PMC10357776; doi:10.1186/s12904-023-01225-1)
Supplement: Supplementary file 5 — Additional File 5: The Australian Carer Toolkit for Advanced Disease: Survey. [file 12904_2023_1225_MOESM5_ESM.pdf]

CARERHELP WEBSITE USER TESTING REVIEW FORM

Website Review Instructions

We have completed the initial development of the CarerHelp website. We are continuing to modify and review content as we get a final copy from the graphic designer and as we complete initial proofing of the site.

We welcome your involvement in this website review and feedback process. Please go to the CarerHelp website and explore the site. Then we invite you to provide comments and feedback by section.

As the site is not yet publicly available, you will need to go the interim splash page at https://www.carerhelp.com.au/.

Step 1. On the top right hand side of page you will see a small purple figure next to the magnifying glass.

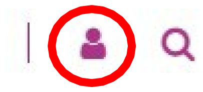

Step 2. Click on this and a login link will appear. Please click on the word Login.

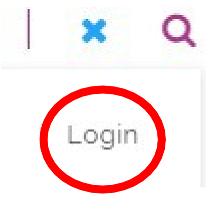

To login, please use the following details:

Username: TBA

Password: TBA

This should open the site for you. At the top of the page you should now have a menu. Click on "Home" and you will be taken to the home page of the new site. If you have any problems, please contact Eric Yang by email: Eric.yang@flinders.edu.au

Response 1: CarerHelp home page

Please answer the following questions:

1. Reading the introductory text on the home page, does it provide you with sufficient information to know what this resource is about?

2. Did you notice the grey boxes at the bottom of the page? Which of these boxes would you choose to click on? And why?

3. Did you notice the 3 straight lines in the top right hand corner? Do you know what they mean and how to use them?

4. We would like to hear further feedback on the home page, in particular any comments on the suitability of the language and the effectiveness of the navigation.

5. We would like to hear your views on the general structure. There are seven main sections in the CarerHelp website – four Carer Pathways (Being an End of Life Carer, Getting prepared, Caring for the Dying, After Caring), Carer Voices, Carer Library, and About the Project. Did you find the content you expected in each section?

Response 2: Carer Pathways (Navigation page)

This page provides an overview of different time points for the carer - from recognising the context is end of life, to life after caring and death. We would like your feedback on some specific matters and more generally.

1. Is it helpful to provide specific resources for different stages?

2. Do the different pathways make sense to you and could you choose the most appropriate pathway for your needs?

3. Is it important that you can download the different pathways rather than only finding them in the website?

Response 3: Comments on the individual Carer Pathways

We do not expect that you will go through each resource and all the modules in each Carer Pathway page but if there are specific pages about which you wish to provide feedback, we would welcome your comments. (Please include the page URL as well as the page title - it will make it easier for us to make corrections to the right page). Please make general and specific comments in the sections below.

Carer Pathway 2 (Being an End of Life Carer): Comments in general or on chosen pages within this pathway

Carer Pathway 3 (Being Prepared): Comments in general or on chosen pages within this pathway

Carer Pathway 4 (Caring for the Dying): Comments in general or on chosen pages within this pathway

Carer Pathway 5 (After Caring): Comments in general or on chosen pages within this pathway

Response 4: Preference for Page Layout

We would now like you to comment on which of two layout options you prefer. We have produced an alternate version of the page for Carer Pathway 2. The content remains the same but we have changed the page presentation.

You can see this page at [Insert URL]

1. Which do you prefer and why?

Response 5: Carer Voice

This section shares stories of carers who have been involved in the project. Please provide feedback on this section and whether you found it helpful.

Response 6: Carer Library

This section includes quality appraised websites, information and resources that can help in providing care for someone who is approaching the end of their life.

1. Did the categories make sense to you, that is, could you find what you were looking for?

2. Is there important information that is missing?

Response 7: About CarerHelp

This page provides information about the project and who is involved. Please provide feedback on this section in general or on specific pages.

Response 8: Any Other Comments

The CarerHelp project team appreciates your time and we would like to thank you for your feedback on the website.

Please forward the completed form to Jennifer.tieman@flinders.edu.au by 5pm on Monday 2 September 2019.
