# Supplementary material for: Who cares for the carers? carerhelp: development and evaluation of an online resource to support the wellbeing of those caring for family members at the end of their life
Source: BMC Palliat Care. 2023 Jul 20;22:98. doi: 10.1186/s12904-023-01225-1 (PMC10357776; doi:10.1186/s12904-023-01225-1)
Supplement: Supplementary file 6 — Appendix 1. Interview and Focus Group Participants [file 12904_2023_1225_MOESM6_ESM.pdf]

The Australian Carer Toolkit for Advanced Disease: Survey

You are invited to take part in this research project, which is called 'The Australian Carer Toolkit for Advanced Disease'. You have been invited because you are involved in the care of a relative, partner or friend who has been diagnosed with an advanced disease or you are a health professional that works with patients with advanced disease.

The research project aims to improve the information and resources available to people who are involved in the care of a relative, partner or friend who has been diagnosed with an advanced disease by developing a website. The website we are developing, called **Carer Help**, includes information, resources and avenues for further support which are relevant for the majority of people caring for someone with an advanced disease. As part of the development process we are inviting health professionals and family members involved in the care of someone with advanced disease to review our draft website.

If you would like to participate in reviewing the draft website, please follow the instructions in the email to login to the draft website. Please take as much time as you like looking at the content of the website. When you are ready to do the survey, please complete the paper version of the survey. The survey is very brief and will take about 5 minutes to complete.

By completing the survey and submitting it, you are giving your consent for us to use your survey responses for our research. Your responses are anonymous and you cannot be identified in any way. Once you complete the survey you will not receive any further correspondence from us. However, if you would like to receive a summary of the results of this survey, then please send an email to "kristina.thomas@svha.org.au', and a summary of the results will be emailed back to you at the end of the study (December 2019).

This research project is being conducted by the Centre for Palliative Care, St Vincent's Hospital Melbourne. It is funded by a grant from the Commonwealth Department of Health.

Further information

All data will be stored securely in a password protected file in the research offices at the Centre for Palliative Care, St Vincent's Hospital Melbourne in accordance with National Health and Medical Research Council requirements. Once the analysis of the research data has been completed the data will be kept for 7 years following publication in keeping with the hospital research and ethics procedures.

The research team may choose to publish these results in academic journals and present them at both national and international medical conferences. In any publication, information will be provided in such a way that you cannot be identified.

All research in Australia involving humans is reviewed by an independent group of people called a Human Research Ethics Committee (HREC). The ethical aspects of this research project have been approved by the HREC of St Vincent's Hospital (Melbourne).

This project will be carried out according to the *National Statement on Ethical Conduct in Human Research (2007)*. This statement has been developed to protect the interests of people who agree to participate in human research studies.

If you want any further information concerning this project or if you have any problems which may be related to your involvement in the project (for example, feelings of distress), please contact a member of the research team.

The principal investigator responsible for this project is:
Professor Peter Hudson, Director, Centre for Palliative Care 03 9416 0000

The project officer is:
Dr Kristina Thomas, Senior Research Fellow, Centre for Palliative Care 03 9416 0000

If you have any complaints about any aspect of the study or the way in which it is being conducted, or questions about your rights as a research participant, then you may contact the Executive Officer Research at St Vincent's Hospital (Melbourne) on Telephone: (03) 9231 3930.

Survey – Carer Help website

Please view as much of the Carer Help website as you like and then aswer the following questions.

1. Are you a:

    ☐ health professional, please describe main discipline ______________________

    ☐ family member, family carer, or friend

    ☐ volunteer

    ☐ other, please describe______________________

Which section/s did you look at?	Not at all	Somewhat	Quite a bit	Very
2. How helpful was the information provided in Carer Help?				
3. How new was the information provided in the Carer Help?				
4. How accurate was the information provided in the Carer Help?				
5. How relevant was the information provided in the Carer Help?				
6. How easy to read was the information provided in the Carer Help?				
7. Was the information presented in an appealing way?				
8. How useful were the videos?				

9. Would you refer someone to Carer Help?

    ☐ Yes

    ☐ No, why not ____

10. Overall please rate Carer Help from 1 to 10 (1=Terrible, 10=Excellent)

Terrible 1_____2_____3_____4_____5_____6_____7_____8_____9_____10 Excellent

11. What did you like most about Carer Help? ____________________

____

____

12. What did you like least about Carer Help? ____________________

____

____

13. What was missing from Carer Help? ____________________

____

____

14. If you are a health professional, how would you use Carer Help with your clients/patients and families? ____________________

____

____

15. If you are a family member or carer, how would you like to find out about Carer Help?

____

____

____

16. How should we promote Carer Help? Who should we promote it to? Where should we advertise?

____

____

____

17. Anything else you would like to add? ____________________

____

____

____

____

____

____

____
